# Safety Signals Enable Single-Session Active Avoidance Paradigm and Expose Threat Generalization in Tuberous Sclerosis Complex

**DOI:** 10.1523/ENEURO.0123-26.2026

**Published:** 2026-09-04

**Authors:** Andrew Gallagher, Amber Victoria Wilson, Saheed Lawal, Harshil Sanghvi, Javed Iqbal, Matthew Dickinson, Brandon Li, Daniel Adolfo Llano, Prerana Shrestha

**Affiliations:** ^1^Graduate Program in Neuroscience, Stony Brook University, Stony Brook, New York 11794; ^2^Department of Neurobiology & Behavior, Stony Brook University, Stony Brook, New York 11794; ^3^Simons STEM Scholars Program, Stony Brook University, Stony Brook, New York 11794; ^4^Graduate Program in Computer Science, Stony Brook University, Stony Brook, New York 11794; ^5^Department of Molecular and Integrative Physiology, University of Illinois at Urbana-Champaign, Illinois 61801; ^6^Beckman Institute for Advanced Sciences and Technology, University of Illinois at Urbana-Champaign, Illinois 61801; ^7^Renaissance School of Medicine, Stony Brook University, New York 11794

**Keywords:** active avoidance, medial prefrontal cortex, memory consolidation, oxytocin receptor neurons, threat-safety discrimination, tuberous sclerosis complex

## Abstract

Animals must flexibly discriminate between threat and nonthreat to deploy adaptive defensive strategies. We introduce a single-session differential signaled active avoidance paradigm that temporally dissociates acquisition, consolidation, and retrieval of instrumental avoidance memory. Interleaving a behaviorally noncontingent neutral cue (CS−) with a threat-predictive, behaviorally contingent cue (CS+) enhanced long-term memory without altering acquisition, demonstrating that differential contingency structure selectively reinforces memory consolidation rather than influencing learning performance. Naive mice of either sex inferred contingencies within a single session, and discrimination achieved during training predicted retrieval precision. However, discrimination operated within defined boundary conditions: Extended training or elevated threat intensity destabilized cue specificity and promoted persistent avoidance generalization. Under high-threat conditions, freezing and shuttling coemerged as complementary defensive responses, indicating a shift from precise cue-based encoding to a generalized defensive state. Remote retrieval recruited oxytocin receptor-expressing cells in the medial prefrontal cortex and activated mTORC1-dependent translational signaling, implicating protein synthesis in maintenance of discriminative avoidance memory. In a tuberous sclerosis complex mouse model with Tsc2 haploinsufficiency in oxytocin-responsive cells, males displayed intact acquisition but generalized avoidance at both recent and remote time points, a deficit not rescued by additional training. These findings identify oxytocin-modulated translational control as a molecular gate stabilizing threat-safety discrimination and show that disruption of this axis—by excessive threat or reduced Tsc2 gene dosage—biases memory toward pathological generalization, providing a mechanistic framework for safety-learning deficits in neurodevelopmental and anxiety-related disorders.

## Significance Statement

Adaptive behavior requires distinguishing between cues that signal threat and safety. Here we introduce a single-session differential signaled active avoidance paradigm that separates acquisition, consolidation, and retrieval of avoidance memory. Interleaving a neutral safety cue with a threat cue strengthens long-term memory consolidation without affecting learning during training. However, excessive threat or overtraining destabilizes cue specificity and promotes avoidance generalization. Remote retrieval engages oxytocin receptor-expressing neurons in the medial prefrontal cortex and activates mTORC1-dependent translational signaling. In a Tsc2 haploinsufficiency model, disruption of this pathway produces persistent avoidance generalization despite intact learning. These findings identify oxytocin-modulated translational control as a mechanism stabilizing threat-safety discrimination and provide a framework for understanding maladaptive fear generalization in neurodevelopmental and anxiety disorders.

## Introduction

Animals navigating dynamic environments must continuously discriminate between threat and safety to deploy appropriate defensive strategies. Defensive behaviors span reactive responses such as freezing and passive avoidance and proactive responses including active escape or avoidance (Blanchard and Blanchard, 1969; Anderson and Adolphs, 2014). Threat imminence theory posits that defensive reactions scale with proximity and controllability of danger, shifting from freezing to flight as threats become imminent (Fanselow and Lester, 1988). Yet when threats are predictable and controllable, adaptive behavior requires more than reflexive responding: animals must flexibly suppress defensive reactions during nonthreat cues while rapidly engaging goal-directed avoidance when danger-predictive cues arise (LeDoux et al., 2017; Evans et al., 2019). Effective threat regulation therefore depends on precise cue discrimination—minimizing unnecessary defensive expenditure during safety to conserve metabolic resources while enabling swift, strategic action when harm is signaled. Disruption of this discrimination process is a defining feature of anxiety disorders, post-traumatic stress disorder, and neurodevelopmental conditions marked by emotional dysregulation, where exaggerated threat generalization and impaired safety signaling drive persistent maladaptive behavior (Woody and Rachman, 1994; Parsons and Ressler, 2013; Cooper et al., 2018; Di Lorenzo et al., 2021; Keefe et al., 2022).

Despite its ethological relevance, instrumental active avoidance remains comparatively understudied at the mechanistic level. Most existing paradigms require multiday training and performance criteria before memory assessment (Krypotos et al., 2018; Diehl et al., 2019; Ali et al., 2024), resulting in overlap between acquisition, consolidation, and retrieval phases. This temporal entanglement has limited circuit-level dissection of avoidance memory relative to single-session pavlovian threat conditioning, which has enabled precise interrogation of consolidation-dependent plasticity (LeDoux, 2000; Shrestha and Klann, 2022). Yet in real-world settings, a single aversive encounter can produce enduring avoidance behaviors. To model this learning dynamic, we developed a single-session differential signaled active avoidance (DSAA) paradigm in which a threat-predictive cue (CS+) requiring instrumental shuttling is interleaved with a neutral safety cue (CS−). This structure permits temporal separation of memory phases while introducing an explicit safety signal that constrains threat prediction. The DSAA paradigm builds upon the traditional signaled active avoidance (SAA) framework, historically influenced by the two-factor “fear-avoidance” theory. This theory posits that reduction of fear elicited by the pavlovian conditioned stimulus (CS) serves as the primary reinforcer driving the initial avoidance learning (Mowrer and Lamoreaux, 1946; Mowrer, 1960; LeDoux et al., 2017; Cain, 2019).

Here we show that incorporation of a safety cue enables robust single-session consolidation of active avoidance memory without altering acquisition. Discrimination achieved during training predicts long-term memory (LTM) precision, and naive animals rapidly infer contingencies without prior instruction. However, stimulus specificity operates within a defined dynamic range: excessive reinforcement or elevated threat intensity destabilizes discrimination and promotes persistent threat generalization. At the circuit level, remote retrieval recruits oxytocin receptor-expressing cells (OTRCs) in the medial prefrontal cortex (mPFC) and engages mammalian target of rapamycin complex 1 (mTORC1)-dependent translational signaling, implicating protein synthesis in discriminative avoidance memory. These observations converge with prior work: prefrontal OTR-expressing cells disinhibit layer II/III pyramidal neurons and thereby constrain anxiety- and stress-related responses (Li et al., 2016; Tabaka et al., 2025); oxytocinergic modulation of limbic–prefrontal circuits promote discrimination between threat- and safety-predictive stimuli (Triana-Del Rio et al., 2022), and Tsc2 haploinsufficiency in these cells produces sex-specific anxiety phenotypes and disrupted innate avoidance (Tabaka et al., 2025). Together, these lines of evidence generate a specific prediction: degrading OTR-cell function should impair learned threat-safety discrimination and bias behavior toward generalization. Testing this directly in OTR.Tsc2 cHET mice, we identify a male-specific impairment in safety cue-dependent discrimination, characterized by generalized avoidance despite intact acquisition. Together, these findings establish DSAA as a sensitive single-session assay for dissecting the neural mechanisms of active avoidance and reveal oxytocin-modulated translational control as a molecular gate that stabilizes threat-safety discrimination and constrains its pathological generalization.

## Materials and Methods

### Mice

Mice were provided with food and water *ad libitum* and were maintained in a 12/12 h light/dark cycle at Stony Brook University or University of Illinois at Urbana-Champaign at stable temperature (78°F) and humidity (40–50%). All mice were backcrossed to C57Bl/6J strain for at least five generations. Both male and female mice, aged 3–6 months, were used in all experiments. Floxed Tsc2 mice were kindly provided by Dr. Michael Gambello (Emory University). OTR Cre BAC transgenic mice (founder line #ON66) were generated by GENSAT and kindly provided by Dr. Nathaniel Heintz (The Rockefeller University). Wild-type C57Bl/6J mice (stock #000664) were purchased from Jackson Laboratory. All procedures involving the use of animals were performed in accordance with the guidelines of the National Institutes of Health and were approved by the University Animal Welfare Committee of Stony Brook University and University of Illinois at Urbana-Champaign.

### Genotyping

OTR-Cre mice were genotyped using the forward primer (5′-CCG GTG AAC GTG CAA AAC AGG CTC TA-3′) and reverse primer (5′-CTT CCA GGG CGC GAG TTG ATA GC-3′). PCR amplification was performed under the following thermocycler conditions: 95°C for 5 min; 95°C for 30 s, 63°C for 45 s, and 72°C for 45 s (30 cycles), followed by 72°C for 10 min and hold at 4°C. A 408 bp band, present in the positive control and absent in negative controls, was considered indicative of the Cre genotype. Tsc2 floxed mice were genotyped using a forward primer (5′-GCA GCA GGT CTG CAG TGA AT-3′), reverse primer 1 (5′-CCT CCT GCA TGG AGT TGA GT-3′), and reverse primer 2 (5′-CAG GCA TGT CTG GAG TCT TG-3′). PCR conditions were as follows: 94°C for 2 min 50 s; 94°C for 30 s, 65°C for 1 min, and 68°C for 1 min (10 cycles), followed by 94°C for 15 s, 60°C for 1 min, and 72°C for 1 min (28 cycles), with a final extension at 72°C for 7 min and hold at 4°C. A 390 bp band corresponded to the floxed allele, whereas a 320 bp band indicated the wild-type allele.

### Stereotaxic surgeries

Mice were anesthetized with the mixture of ketamine (100 mg/kg) and xylazine (10 mg/kg) in sterile saline (intraperitoneal injection). Adequate sedation was determined by a lack of gentle toe pinch withdrawal reflex. A lubricant eye ointment (GenTeal Tears; Alcon Laboratories) was applied on the eyes to prevent ocular dehydration. Visual monitoring of respiration occurred periodically throughout surgery to confirm survival. Stereotaxic surgeries were carried out inside a Class II A2 biosafety cabinet (LabRepCo, #NU-677-500) on the Kopf stereotaxic instrument (model number 942), which was equipped with a Nanojector UMP3TA (WdI). Viral vectors were injected intracranially using 2.0 µl Neuros syringe (Hamilton, #65459-02) at 1 nl/s. Before removing the needle, an interval of 10 min was allotted for viral diffusion. Upon completion of intracranial injections, the incision of the scalp was closed using a tissue adhesive (3M Vetbond). Normothermia during surgery was maintained at 36.6°C by resting the mouse on a covered heating pad (7 × 7 cm) connected to a rodent warmer console (Stoelting, #53800 M). To prevent somatic dehydration, each mouse was subcutaneously injected with 500 µl of sterile saline before surgery and further provided with hydrogel (ClearH2O, #70-01-5022) in the recovery cage. General monitoring and analgesia using 3 mg/kg subcutaneous ketoprofen (Zoetis) were provided up to 3 d postsurgery. To label oxytocin receptor-expressing neurons in the mPFC, 200 nl of AAV8.hSyn.DIO.GFP (1.00 × 10^13^ GC/ml) was injected into the stereotaxic coordinates [−2.00 mm anterior posterior (AP), ±0.25 mm mediolateral (ML), and −1.60 mm dorsoventral (DV)] of either OTR Cre+/− wild-type mice. AAV8-hSyn-DIO-EGFP was a gift from Bryan Roth (Addgene plasmid #50457; http://n2t.net/addgene:50457; RRID:Addgene_50457).

### Auditory brainstem responses

Wild-type C57Bl/6J male and female mice were anesthetized with intraperitoneal ketamine (100 mg/kg), xylazine (6 mg/kg), and acepromazine (3 mg/kg). After achieving surgical anesthesia, subdermal electrodes (active, recording, and ground) were placed at the vertex, behind the left ear, and behind the right ear, respectively. Tone bursts (5 ms duration; 1 ms rise/fall, 3 ms plateau) were presented at 21 Hz. A 3 kHz stimulus was delivered free field via an AS03608AS-R speaker (PUI Audio) positioned 5 cm ipsilateral to the recording ear, while 7.5, 15, and 30 kHz stimuli were delivered via a TDT ES1 speaker positioned 5 cm from the interaural midline. Speakers were driven by TDT System 3 hardware with RPvdsEx software and calibrated using a Svantek 977 Sound and Vibration Level Meter and Larson Davis CAL200 calibrator. Auditory brainstem responses (ABRs) were acquired using TDT RA4PA Medusa preamps, bandpass filtered (500–3,000 Hz), and averaged over 512 sweeps within a 30 ms window. Thresholds were determined using a bracketing approach from 90 to 10 dB SPL. Coarse 10 dB decrements identified a response bracket, followed by 2 dB steps to define threshold as the lowest level producing identifiable waves I–V (0–10 ms), ≥100 nV amplitude, consistent morphology at +2 dB, and absence at −2 dB.

### Behavior

All behavior sessions were conducted during the light cycle (7:00 A.M.–7:00 P.M. EST). Both male and female mice were included in behavior experiments. Behavior experiment data were collected by experimenters that were blind to the experimental conditions and genotype. Following weaning, mice were group-housed with up to three sex-matched littermates in standard cages (8 × 13 × 5 in) fitted with corn cob bedding and Enviro-dri enrichment. Acclimation of the mice in the behavior room lasted at least 30 min prior to testing. A white noise machine (Yogasleep Dohm; 58 dB at a distance of 6 ft from the machine) was used to blunt any environmental noise that may induce confounding anxiety. All items in the behavior equipment were cleaned with 30% ethanol unless otherwise stated.

#### Data acquisition

Behavioral experiments were performed using the Habitest Linc System (H01-01 Power Base and H02-08 Linc Boxes; Harvard Apparatus) controlled by the Graphic State 4 software (Coulbourn Instruments), which regulated delivery of footshock, light stimuli, and auditory tones. Graphic State 4 also recorded shuttling behavior throughout each session. Freezing during DSAA training and memory retrieval was quantified using the FreezeFrame 4 software (Actimetrics), which captured video and automatically scored freezing responses. Two distinct environmental contexts (Contexts A and B) were used for the SAA and DSAA paradigms. Context A consisted of a chamber (29 cm W × 23 cm D × 20 cm H) equipped with a white house light and metal shock grid floor, cleaned with 30% ethanol, and lacking striped wall panels. Context B was a chamber of similar dimensions but contained infrared illumination, striped wall panels, a white plexiglass platform floor, vanilla-scented paper bedding and was cleaned with 30% isopropanol to provide distinct visual, tactile, and olfactory cues. Chambers were housed within a sound-attenuating isolation cubicle (30 W × 19H × 18D; H10-24 and H10-24A, Coulbourn Instruments). Footshocks were delivered via a Precision Animal Shocker (H13-15, Coulbourn Instruments), and auditory stimuli were presented programmatically using an H12-01M speaker module coupled to an H12-07 seven-tone audio cue.

#### Single-tone SAA

Training: Mice were acclimated to Context A for 120 s before training commenced. Training consisted of 11 CS+ presentations (7.5 kHz pulsed tone; 0.2 s on, 0.2 s off) delivered with a variable intertrial interval (ITI; 20–90 s, mean 45 s). Avoidance was defined as a shuttle response during the CS+, which immediately terminated the tone and prevented footshock delivery. Shuttling is the movement from one chamber to the adjacent chamber through an unobstructed doorway separating the two chambers. The response was not direction- or chamber-specific but rather constituted the instrumental action of crossing between compartments. Failure to shuttle within the 15 s CS+ resulted in delivery of a 0.2 mA footshock [unconditioned stimulus (US)] for 0.5 s. Shuttling during the shock period was classified as an escape response.

Recent LTM test: 24 h after training, mice were acclimated to Context B for 120 s prior to testing. They then received five CS+ presentations with a variable ITI (20–90 s, mean 45 s). Context B was defined by a white Plexiglas floor, striped walls, infrared illumination, vanilla-scented bedding, and 30% isopropanol as the olfactory cue. Trials were nonreinforced. Avoidance was measured as shuttling during the CS+, which terminated the tone. No footshock was delivered during the LTM test, even if the animal failed to avoid.

#### Pavlovian trial

The pavlovian conditioning trial consisted of an unavoidable footshock delivered immediately following the CS+ in Context A. The CS+ was a 7.5 kHz pulsed tone (0.2 s on, 0.2 s off) presented for 15 s at 40% amplitude (∼60 dB) through a speaker mounted on the far wall of each chamber. Immediately after CS+ termination, a 0.2 mA steady footshock was delivered for 0.5 s. The CS− was a 3 kHz continuous tone presented at 60% amplitude (∼80 dB) for 15 s. The CS− was delivered 2 min after the US.

#### DSAA

*Training*: Mice were acclimated to Context A for 120 s before training began. Training consisted of 11 CS+ presentations (7.5 kHz pulsed tone; 0.2 s on, 0.2 s off; 60 dB; ≤15 s duration) delivered with a variable ITI (20–90 s, mean 45 s). Avoidance was defined as a shuttle response during the CS+, which immediately terminated the tone and prevented footshock delivery. Shuttling was defined as movement from one chamber to the adjacent chamber through an unobstructed doorway separating the two compartments. This response was not direction- or chamber-specific but rather represented the instrumental act of crossing between compartments. Failure to shuttle within the 15 s CS+ resulted in delivery of a 0.2 mA footshock (US) for 0.5 s. Shuttling during the shock period was classified as an escape response. In addition, 11 CS− presentations (3 kHz continuous tone; 80 dB; ≤15 s duration) were interleaved with CS+ trials. The CS− was never reinforced in any animal, independent of CS+ performance, so its objective contingency was identical across subjects. The CS− was also separated in time from the avoidance response (AR) by 20–90 s, so that it did not serve as a feedback signal for CS+ avoidance. Shuttling during the CS− was scored as a CS− evoked AR.

*Recent LTM test*: 24 h after training, mice were acclimated to Context B for 120 s prior to testing. They then received five CS+ presentations with a variable ITI (20–90 s, mean 45 s). All trials were nonreinforced. CS+-evoked avoidance was measured as shuttling during the CS+, which terminated the tone. No footshock was delivered during the LTM test, even if the animal failed to avoid. Five CS− presentations were similarly interleaved with CS+ trials. CS+- and CS−-evoked AR were quantified as shuttling during their respective cue presentations, each of which terminated the tone.

*Remote LTM test*: Remote LTM was assessed in Context B 28 d after training. Testing parameters were identical to those used for the recent LTM assessment.

#### US-intensity adjustments during DSAA training

Mice were trained using the standard DSAA protocol, with the exception that the intensity of the US was systematically varied. The standard condition employed a 0.2 mA continuous footshock (medium US). In separate groups, the US intensity was adjusted to 0.1 mA continuous footshock (weak US) or 0.4 mA continuous footshock (strong US).

#### Extended training

Mice were trained using the standard DSAA protocol across 3 consecutive days. Each day, animals were acclimated to Context A for 120 s, followed by 11 interleaved presentations of CS+ and CS−. Only the CS+ maintained the tone-shock contingency, such that failure to shuttle during the CS+ resulted in footshock delivery. Remote LTM was assessed 28 d after the final training session in Context B. The test consisted of five interleaved CS+ and CS− presentations delivered with a variable ITI (20–90 s; mean 45 s).

Note that the naive DSAA group (Fig. 2) and the single-session group (Fig. 3) and the medium US group (Fig. 4) represent a single shared reference cohort, as these conditions are experimentally identical (naive protocol, 0.2 mA US, single training session) and serve as the common comparator against which the priors, extended-training, and US-intensity manipulations are evaluated.

#### Data processing pipeline

To facilitate efficient and reproducible analysis of avoidance conditioning experiments, we developed a fully automated, Python-based computational pipeline for data extraction, behavioral metric computation, and visualization. The workflow integrates timestamped event logs from FreezeFrame (freezing during pavlovian threat conditioning and DSAA) and GraphicState [threat-predictive CS (CS+), safety cue (CS−), and US-evoked responses] to generate structured, analysis-ready datasets. Custom modules (freezeframe.py, graphicstate.py) implement preprocessing steps including column name standardization, epoch segmentation (pre-CS, CS+, CS− for freezing behaviors, and non-CS, CS+, CS− for shuttling responses), metadata integration, and handling of missing data. Task-specific extensions (dsaa_gs.py) were generated to classify avoidance trial outcomes (avoidance, escape, failure), compute spontaneous and CS+ and CS− evoked shuttling, and calculate latency to avoid using precisely parsed CS onset/offset timestamps. On the other hand, dsaa_ff.py was generated to quantify freezing percentages across defined epochs (pre-CS, CS+, and CS−). The pipeline uses an object-oriented modular design built around the FreezeFrame and GraphicState base classes, supporting reusability across experimental paradigms. Automated batch processing was implemented for high-throughput analysis of multiple datasets with minimal manual input. Comprehensive logging and error handling (via logging.info(), logging.warning(), try-except blocks) were also utilized to ensure data integrity and traceability. Dedicated plotting modules (fr_pav_heatmaps.py) were developed to generate standardized visualizations of freezing dynamics, allowing rapid and reproducible figure generation. Together, this custom pipeline provided a scalable and transparent framework for behavioral data analysis in DSAA paradigm.

### Histology

Mice were deeply anesthetized with isoflurane and transcardially perfused with 0.1 M PBS followed by 4% paraformaldehyde (PFA; Electron Microscopy Sciences) in PBS. Brains were removed and postfixed in 4% PFA for 24 h. The PFA is then replaced with 30% sucrose solution for another 24 h. The 40 µm free-floating coronal brain sections containing PFC were collected using Leica Vibratome (VT1000 s) and stored in 1× PBS containing 0.05% sodium azide at 4°C. After blocking in 5% normal goat serum in 0.1 M PBS with 0.1% Triton X-100, brain sections were probed overnight with primary antibodies [chicken anti-EGFP (Abcam #ab13970) and rabbit anti-p-S6 (S235/6; Cell Signaling Technology #4898, Lot #21)]. After washing three times in 0.1 M PBS, brain sections were incubated with Alexa Fluor-conjugated secondary antibodies 1:200 (Thermo Fisher Scientific A32931 Lot #VK314825, A32740 Lot #WC316326) in blocking buffer for 1.5 h at RT and mounted using ProLong Gold Antifade Mountant (Thermo Fisher Scientific #P36930).

### Imaging

Imaging data from immunohistochemistry experiments were acquired using a laser scanning microscopy confocal microscope (Zeiss) with 20× objective lens (with 1× or 2× zoom), and *z*-stacks (∼6 optical sections with 0.563 µm step size) for three coronal sections per mouse from AP bregma +1.98 to +1.78 mm (*n* = 3 mice) were collected. Imaging data were analyzed with ImageJ using the Bio-Formats importer plugin. Maximum projection of the *z*-stacks was generated followed by manual outline of individual cells and mean fluorescence intensity measurements using the drawing and measure tools. Mean fluorescence intensity values for all cell measurements were normalized to the mean fluorescence intensity for controls. In the case of verifying Rapamycin's effect on the mTORC1 pathway in mPFC, field fluorescence of the entire image area was measured, instead of outlining each cell. This was done to prevent underrepresentation of images with fewer labeled cells. Each image was opened with ImageJ, and the six *z*-stacks of the images were subjected to maximum projection with maximum intensity. A rectangle was used to outline the entire perimeter of the image, and the fluorescence was measured. The area and mean intensity of the image were measured.

### Statistics

Statistical analyses were performed using GraphPad Prism 8 (GraphPad software). Data are expressed as mean ± SEM. Data from two groups were compared using two-tailed unpaired Student's *t* test. Multiple group comparisons were conducted using one-way ANOVA, two-way ANOVA, or RM two-way ANOVA with post hoc tests as described in the appropriate figure legend. Statistical analysis was performed with an α level of 0.05. *P* values <0.05 were considered significant.

## Results

### Adding a safety cue improves consolidation and expression of SAA

Consistent with our previously established threat discrimination protocol (Shrestha et al., 2020), we selected 7.5 and 3 kHz tone frequencies to serve as the CS+ and CS−, respectively, in the DSAA paradigm. To exclude the possibility that behavioral differences were driven by unequal tone audibility, we assessed ABRs in male and female C57BL/6 mice. Under anesthesia, hearing sensitivity was quantified by recording brainstem-evoked potentials in response to tone bursts presented at progressively increasing sound intensities, allowing determination of auditory thresholds for each frequency. Both sexes exhibited comparable hearing ranges, with higher thresholds for the 3 kHz CS− tone (∼70 dB) than for the 7.5 kHz CS+ tone (∼45 dB; Fig. 1*A*). Accordingly, we adjusted the stimulus amplitudes such that CS+ and CS− were presented at 60 and 85 dB, respectively, ensuring that both cues were well above threshold and perceived at comparable salience.

**Figure 1.**
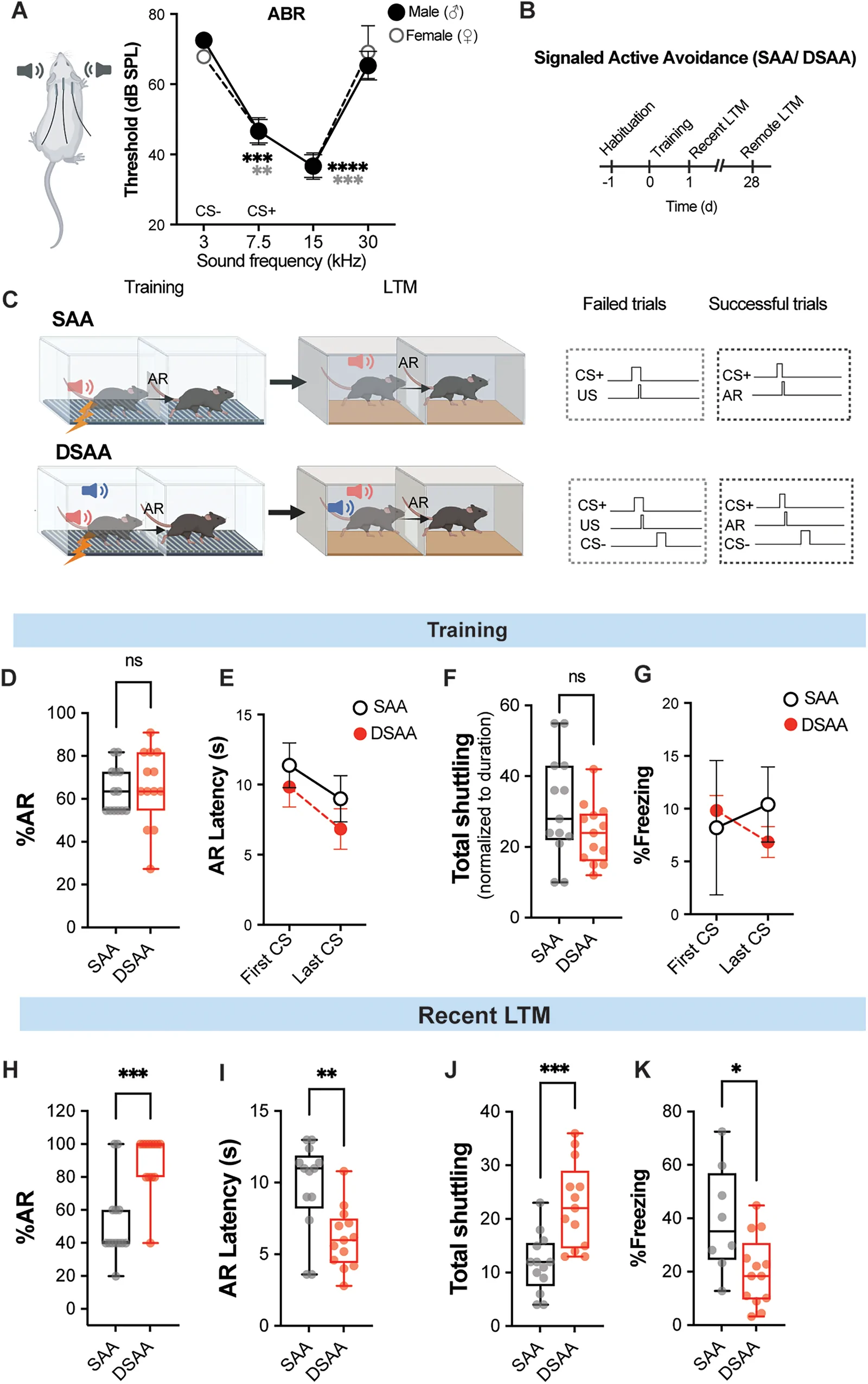
***A, B***, Schematic comparison of conventional SAA and the DSAA paradigm. In SAA, animals learn an instrumental response to a threat-predictive cue (CS+) to avoid the US, without presentation of a safety cue. In contrast, DSAA introduces a behaviorally noncontingent neutral cue (CS−), requiring animals to discriminate between threat (CS+) and safety (CS−). In both paradigms, failed trials result in US delivery immediately following CS+, whereas successful trials consist of an AR during CS+, which terminates the cue and prevents the US. During DSAA training and LTM testing, the CS− follows either the US (failed trials) or the AR (successful trials), respectively. ***B***, ABRs recorded from male and female C57BL/6J mice across a range of sound frequencies (3, 7.5, 15, and 30 kHz). ***C***, The percentage of AR during training was comparable between SAA and DSAA groups. ***D***, Reduction in avoidance latency from the first to the last CS+ presentation was similar across groups. ***E***, Total shuttling behavior (normalized to session duration) and ***F***, CS+-evoked freezing during training did not differ between paradigms. ***G***, During recent LTM testing, DSAA animals exhibited significantly higher avoidance rates relative to SAA, indicating enhanced consolidation and retrieval. ***H***, CS+-evoked avoidance latencies were further reduced in the DSAA group, demonstrating faster execution of the AR. ***I***, Total shuttling (normalized to duration) was increased in DSAA animals. ***J***, Freezing responses remained prominent in SAA but were significantly attenuated in DSAA. Statistical tests: ***B***, ***E***, ***G***, RM two-way ANOVA with Bonferroni’s post hoc test; ***D***, ***F***, and ***H***–***K***, unpaired *t* tests. *p < 0.05; **p < 0.01. Sample sizes: ***B***–***G***, *n* = 6–13 mice per group. *p < 0.05; **p < 0.01; ***p < 0.001; ns, not significant. Further characterization of the DSAA paradigm, including CS+/CS− discrimination, the 11-CS protocol rationale and individual-difference analyses, is provided in Extended Data Figure 1-1.

10.1523/ENEURO.0123-26.2026.f1-1 Figure 1-1 **A)** Characterization of differential signaled active avoidance. Mice were trained with a CS + that terminated in footshock upon failure to avoid and a CS− carrying no shock contingency. Through approximately the fifth CS presentation, avoidance latencies were generalized across the two cues; latencies to CS + and CS− then progressively diverged, reaching significance by CS set 11. **1-1B)** Pearson correlation between percent avoidance responses to the CS- and to the CS+, revealing a continuous generalization gradient that scales with overall avoidance drive. A significant positive correlation is observed (r = 0.71, r² = 0.501, p = 0.0068). **1-C)** Trend-level anti-correlation between discrimination index and percent avoidance responses to the CS + (r = -0.499, r² = 0.249, p = 0.083). **Statistical tests:** 1-1A) RM two-way ANOVA with Bonferroni post-hoc test; 1-1B, C) Pearson correlation test with linear regression. *p < 0.05, **p < 0.01. **Sample sizes:** 1-1A-C) n = 13 mice. *p < 0.05, **p < 0.01, ***p < 0.001; ns, not significant. Download Figure 1-1, TIF file.

Conventional single-tone SAA is characterized by slow acquisition and typically requires multiple days of training. In our implementation, mice received 11 presentations of the CS+ (7.5 kHz, 15 s pulse), each predicting an impending footshock (0.2 mA) that the animal could avoid by shuttling during the tone; no additional auditory cues were provided. To test whether incorporating a neutral auditory tone enhances learning or memory retention, we implemented a DSAA protocol in which the CS+ presentations were interleaved with CS− trials (3 kHz pure tone for 15 s; Fig. 1*B*,*C*). During training, SAA and DSAA mice performed comparably, with no significant differences in percent avoidance or avoidance latency (Fig. 1*D*,*E*). Total shuttling and percent freezing were also similar between groups (Fig. 1*F*,*G*), indicating equivalent baseline locomotor activity and defensive responses. Relative to SAA-trained mice, DSAA-trained mice exhibited superior memory retrieval—higher avoidance rates, shorter avoidance latencies, increased shuttling, and reduced freezing—and outperformed their own first-day training levels (Fig. 1*H*–*K*). These findings suggest that inclusion of a safety cue enhances consolidation of CS+-evoked active avoidance.

The number of CS+ presentations was chosen based on our previously validated threat discrimination protocol, in which three presentations each of the CS+ and CS− within a single session were sufficient to establish a discriminative reactive freezing memory (Shrestha et al., 2020). Under DSAA, however, mice failed to discriminate between the two stimuli through the first five CS+/CS− presentations, despite the tone-shock contingency for the CS+. Discrimination emerged only after >10 presentations: by the 11th presentation, avoidance latencies for the two cue types differed significantly (Extended Data Fig. 1-1*A*). Because the total session duration remained ∼27 min—still within the bounds of single-session DSAA—we adopted the 11-CS protocol for all subsequent studies. This regimen was sufficient to drive robust consolidation while remaining below the extended-training range that we subsequently found to degrade cue specificity.

Because the CS− was never reinforced for any animal, regardless of CS+ performance, its objective contingency was identical across all subjects; it was additionally dissociated in time from the AR by 20–90 s and therefore did not function as a feedback signal. To assess how responding to the two cues related across individuals, we examined the relationship between CS+ and CS− avoidance. Percent avoidance to the CS+ and CS− were positively correlated (Extended Data Fig. 1-1*B*; *r*^2^ = 0.501; *p* = 0.0068**), with the highest CS+ avoiders also showing the highest CS− responding. This pattern indicates that residual responding to the neutral cue covaried with a shared, graded avoidance propensity across the cohort rather than arising from failure to acquire the CS+ contingency—a mechanism that would instead predict elevated or erratic CS− responding among poor CS+ performers. Consistent with this interpretation, the discrimination index showed a trend-level decline as CS+ avoidance increased (Extended Data Fig. 1-1*C*; *r*^2^ = 0.249; *p* = 0.083). Together, these relationships indicate that DSAA yields a graded, individually variable discrimination phenotype, positioning the discrimination index as a sensitive readout of memory precision for the analyses that follow.

### Contingency learning in differential SAA is robust in the absence of prior information

To test whether priors influence the DSAA performance, we implemented a strategy in which mice either received explicit tone-shock contingency information (“priors”) or remained naive (Fig. 2*A*). The priors group received a pavlovian conditioning trial 1 h before the DSAA training. The pavlovian trial consisted of a single presentation of the CS+ (7.5 kHz pulsed tone, 15 s) immediately followed by an unavoidable footshock (US; 0.2 mA, 0.5 s). Within the same trial, the CS− (3 kHz pure tone, 15 s) was presented after a 90 s interval. The priors were added to test whether single-session consolidation requires explicit tone-shock instruction or instead emerges from contingency inference under initial uncertainty. Naive mice did not receive this pavlovian pre-exposure. LTM was assessed at two time points: 24 h after training and again 28 d later in a novel context, to test whether the auditory CS could elicit an instrumental AR in the absence of reinforcement.

**Figure 2. eN-NWR-0123-26F2:**
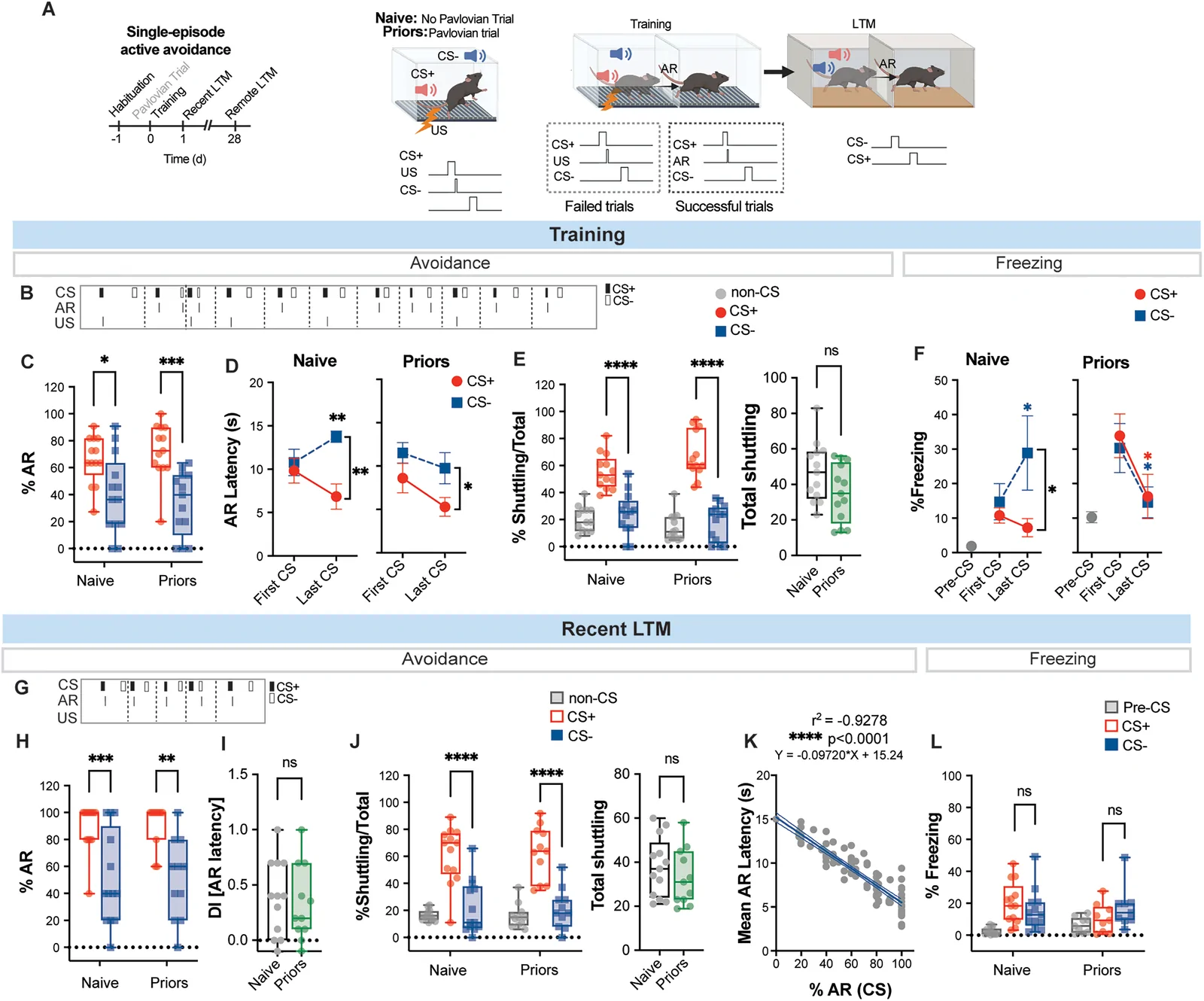
***A***, Schematic of the single-session DSAA paradigm. Mice undergo a single training session, preceded by a pavlovian conditioning trial in the priors group, followed by longitudinal assessment of recent and remote LTM at 24 h and 28 d post-training. Failed trials are defined as trials in which mice do not shuttle during the CS+ and consequently receive the footshock US. Successful trials are those in which mice execute an AR during the CS+, thereby preventing shock delivery. The neutral safety cue (CS−) carries no behavioral contingency. ***B***, Representative event log illustrating the temporal occurrence of conditioned stimuli (CS+ and CS−), AR, and US for a single mouse during DSAA training. Each of the 11 sets of CS+/CS− are separated with dashed lines. ***C***, Box-and-whisker plots showing percent AR to CS+ and CS− during DSAA training in naive and priors groups. ***D***, Learning curves depicting changes in avoidance latency from the first to the last CS presentation for each cue (CS+ and CS−) in naive (left) and priors (right) groups. ***E***, Left, Distribution of shuttling behavior (expressed as percent of total shuttling) across CS+, CS−, and non-CS epochs. Right, Total shuttling during training in naive and priors groups. ***F***, Percent freezing during DSAA training measured during pre-CS, first CS, and last CS epochs in naive and priors groups. ***G***, Representative event log illustrating the temporal occurrence of CS (CS+ and CS−), AR, and US for a single mouse during the DSAA recent LTM test. ***H***, Cue-evoked AR during the recent LTM test. ***I***, Discrimination index (DI) based on avoidance latency for naive and priors groups. ***J***, Left, Shuttling distribution across CS+, CS−, and non-CS epochs during recent LTM testing. Right, Total shuttling during recent LTM in naive and priors groups. ***K***, Pearson’s correlation analysis demonstrating a strong positive correlation between mean avoidance latency and percent AR (training and LTM data pooled across CS+ and CS−; *r*^2^ = 0.9278; ****p < 0.0001). ***L***, Percent freezing during the recent LTM test in naive and priors groups. Statistical analyses: ***C***, ***E*** (left), ***H***, ***J*** (left), ***L***, two-way ANOVA with Bonferroni’s post hoc test; ***D***, ***F***, RM two-way ANOVA with Bonferroni’s post hoc test; ***E*** (right), ***I***, ***J*** (right), unpaired *t* test. Sample size, *n* = 6–13 mice per group. *p < 0.05; **p < 0.01; ***p < 0.001; ****p < 0.0001; ns, not significant. Further characterization of cue-specific discrimination across both the naive and prior-conditioning group is provided in Extended Data Figure 2-1.

10.1523/ENEURO.0123-26.2026.f2-1 Figure 2-1 **A)** Both naïve and prior-conditioning groups exhibited appropriate cue-specific discrimination, reflected by significantly shorter avoidance latencies to the CS + relative to the CS−. **2-1B)** Discrimination indices for cue-specific avoidance latency during recent long-term memory (LTM) testing were positively correlated with discrimination indices measured during training, indicating that training-phase discrimination predicts subsequent memory precision (Pearson correlation: R² = 0.3694; Y = 0.3793X + 0.1328). **Statistical analyses:** 2-1A) Two-way ANOVA with Bonferroni post hoc comparisons. 2-1B) Pearson correlation analysis. **Sample size**: n = 6-13 mice per group. *p < 0.05, **p < 0.01, ***p < 0.001, ****p < 0.0001; ns, not significant. Download Figure 2-1, TIF file.

On the training day, both naive and priors groups exhibited a significantly higher proportion of AR during CS+ presentations compared with CS− (Fig. 2*B*, representative single-session raster plot, *C*). Analysis of avoidance latencies across training revealed similar learning dynamics between groups. In the naive group, avoidance latency decreased across CS+ trials but increased across CS− trials, resulting in a marked enhancement of cue discrimination. The priors group showed modest but significant changes in avoidance latency between early and late trials (Fig. 2*D*). Total shuttling activity and the distribution of shuttling across non-CS, CS+, and CS− epochs did not differ between groups. In both groups, shuttling was highest during CS+ presentations, whereas shuttling during CS− and non-CS epochs remained comparably low (Fig. 2*E*), indicating that group differences in discrimination were not driven by differences in overall locomotor activity. Because passive and active defensive responses are often inversely related, we next examined freezing behavior across training. In the naive group, freezing increased from first to last CS− trials but remained stable across CS+ trials. In contrast, the priors group exhibited a steady decline in freezing across training for both CS+ and CS− (Fig. 2*F*). We interpret this as follows: in the pavlovian trial to which the priors group was subjected, each CS+ was followed shortly by the unavoidable US and, 120 s later, by CS−; this arrangement established a secondary CS−/footshock association that produced a robust pavlovian freezing response to the first CS presentation during subsequent avoidable DSAA trials, which diminished for both CS+ and CS− over the course of training as the animals learned to avoid.

During the recent LTM test conducted in a novel context—distinguished by olfactory, visual, and somatosensory cues—both naive and priors groups exhibited a significantly higher proportion of AR during CS+ relative to CS−, despite the absence of reinforcement (Fig. 2*G*, representative raster plot, *H*). This produced a robust discrimination index based on avoidance latency between CS+ and CS− (Extended Data Fig. 2-1*A*), alongside a comparable distribution of shuttling across CS and non-CS epochs in both groups (Fig. 2*I*). Moreover, the percentage of AR was strongly correlated with mean avoidance latency for both CS types (Fig. 2*J*), indicating that across training and memory retention testing, animals executed the instrumental AR with progressively shorter latencies as cue–outcome associations were consolidated. In addition, discrimination indices measured during training were strongly positively correlated with those observed during the recent LTM test (Extended Data Fig. 2-1*B*), indicating that successful avoidance performance during memory retrieval directly reflected learning achieved during the training session. Freezing responses were minimal in both naive and priors groups, and no clear discrimination was observed between CS+ and CS− based on freezing behavior (Fig. 2*L*).

Together, these results demonstrate that robust single-session discrimination and its faithful retrieval at the recent LTM test do not require explicit tone-shock instruction but emerge equally well from contingency inference under initial uncertainty.

### Extended training impairs stimulus discrimination during remote retrieval of cued DSAA memory

We next asked whether additional training modifies the strength or specificity of DSAA memory at a remote time point—a boundary condition with direct relevance to compulsive, rigid avoidance. To test whether further reinforcement sharpens cue specificity or, conversely, degrades it, mice received two additional days of reinforced DSAA training in the same context (Context A) and were designated the “Extended training” group (Fig. 3*A*). Across these sessions, mice showed increased AR and reduced response latencies to both the CS+ and CS− (Fig. 3*B*,*C*) while maintaining comparable latency-based stimulus discrimination (Fig. 3*D*). The proportional distribution of shuttling across CS+, CS−, and non-CS epochs remained stable, although total shuttling increased on the second day, consistent with heightened defensive engagement and enhanced contextual responding (Fig. 3*E*,*F*). Mice froze more to the neutral cue (CS−) on the first training day, but freezing was minimal on subsequent days, reducing the freezing-based discrimination index (Extended Data Fig. 3-1*A*,*B*).

**Figure 3. eN-NWR-0123-26F3:**
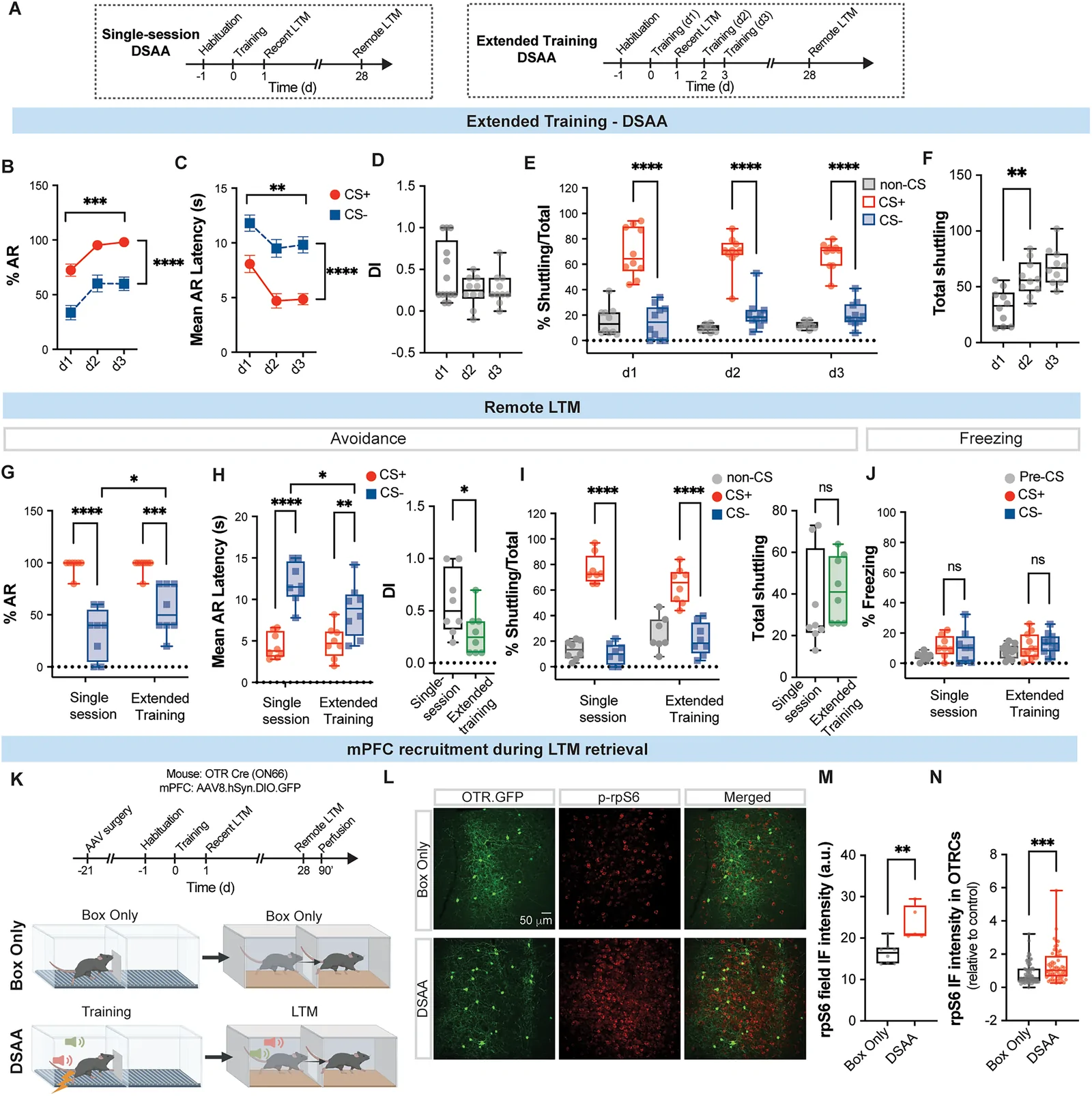
***A***, Schematic comparing the single-session DSAA protocol with the extended-training DSAA protocol, which included two additional days of reinforced training. ***B***, Following two additional days of extended training, naive DSAA mice exhibited enhanced avoidance performance to both CS+ and CS−. ***C***, Mean AR latencies progressively decreased across training days for both auditory cues. ***D***, Box-and-whisker plots demonstrate that the discrimination index for avoidance latency remained stable across the three training sessions. ***E***, The distribution of shuttling behavior (expressed as percentage of total shuttling) across CS+, CS−, and non-CS epochs remained consistent over training. ***F***, Total shuttling increased from Day 1 to Day 2 and plateaued from Day 2 to Day 3. ***G***, Box-and-whisker plots depicting percent AR to CS+ and CS− in single-session versus extended-training groups during remote LTM. ***H***, Left, Mean avoidance latencies to CS+ and CS− during remote LTM testing for single-session and extended-training groups. Right, Discrimination index, showing a significant reduction in extended-training mice compared with single-session controls. ***I***, Left, Distribution of shuttling behavior (expressed as percentage of total shuttling) across CS+, CS−, and non-CS epochs for single-session and extended-training groups. Right, Total shuttling during the remote LTM test. ***J***, Percent freezing during pre-CS, CS+, and CS− epochs for single-session and extended-training groups, demonstrating minimal freezing across conditions. ***K***, Schematic illustrating labeling of OTRCs in the mPFC, DSAA training, and longitudinal LTM testing. The tissue was collected 30 min after memory retrieval on Day 28. Box-only controls were exposed to the training and testing contexts without cue or shock presentation. ***L***, Representative images of phosphorylated ribosomal protein S6 (p-rpS6) expression in OTRCs from box-only and DSAA-trained mice. Scale bar, 50 µm. ***M***, Quantification of p-rpS6 immunofluorescence (IF) field intensity. ***N***, Quantification of p-rpS6 IF within OTRCs, demonstrating increased activation in OTRCs of DSAA mice relative to box-only controls. Statistical analyses: ***B***, ***C***, RM two-way ANOVA with Bonferroni’s post hoc test; ***D***, ***F***, one-way ANOVA with Bonferroni’s post hoc test; ***E***, ***G***, ***H*** (left), ***I*** (left), ***J***, two- way ANOVA with Bonferroni’s post hoc test; ***H*** (right), ***I*** (right), ***M*** (left), and ***M*** (right), unpaired *t* test. Sample size: ***B–J***, *n* = 6–8 mice per group; ***M***, *n* = 5–8 sections from three mice per group; ***N***, *n* = 59–87 neurons from three mice per group. *p* < 0.05; *p* < 0.01; *p* < 0.001; *p* < 0.0001; ns, not significant. Further characterization of differential freezing response to CS− is provided in Extended Data Figure 3-1.

10.1523/ENEURO.0123-26.2026.f3-1 Figure 3-1 A) Differential freezing response to the CS- was evident on the first day of DSAA training; however, freezing declined to baseline levels on days 2 and 3. **3-1G)** The freezing discrimination index significantly decreased from day 1 to day 2, consistent with reduced cue-specific freezing over time. **Statistical analyses:** 3-1A) RM Two-way ANOVA with Bonferroni post hoc test; 3-1B) One-way ANOVA with Bonferroni post-hoc test. **Sample size:** 3-1A, G) n = 10 mice per group. p < 0.05, p < 0.01, p < 0.001, p < 0.0001; ns, not significant. Download Figure 3-1, TIF file.

When tested for remote LTM on Day 28 in a distinct context (Context B), both naive and extended-training mice exhibited robust AR to the CS+ and retained discrimination between the two conditioned stimuli (Fig. 3*G*). However, extended-training mice showed elevated AR and reduced latency to the CS−, resulting in a significant reduction in the discrimination index (Fig. 3*H*). Extended training also altered the temporal distribution of shuttling, yielding more similar levels of responding across CS+, CS−, and non-CS epochs, whereas this distribution remained preserved in naive mice. Importantly, extended training did not induce generalized hyperactivity, as total shuttling remained unchanged (Fig. 3*I*). Freezing responses remained minimal in both groups, and no CS-specific freezing discrimination was observed (Fig. 3*J*).

The mPFC has been implicated in the expression of both passive and active avoidance behaviors (Santini et al., 2004; Moscarello and LeDoux, 2013; Bravo-Rivera et al., 2014; Rosas-Vidal et al., 2018; Gu and Johansen, 2025). Within this region, OTRCs are proposed to disinhibit layer II/III pyramidal neurons, thereby enhancing local network excitability and constraining anxiety- and stress-related responses (Shrestha et al., 2015; Li et al., 2016; Tabaka et al., 2025). Oxytocinergic neuromodulation of limbic–prefrontal circuits is thought to promote discrimination between stimuli that predict threat versus safety (Sabihi et al., 2014; Olivera-Pasilio and Dabrowska, 2020; Triana-Del Rio et al., 2022). Consistent with this view, oxytocin signaling in the central amygdala has been shown to facilitate a transition from passive freezing to active escape in response to imminent but escapable threats while attenuating reactivity to more distal threats (Terburg et al., 2018). However, whether oxytocin modulates prefrontal network activity during SAA remains unknown.

To determine whether DSAA memory retrieval engages prefrontal OTRCs, we used OTR:Cre transgenic mice (ON66; Nakajima et al., 2014) and injected a cre-dependent viral vector expressing GFP into the prelimbic cortex (Fig. 3*K*). The brain tissue was collected 30 min after remote DSAA memory retrieval. Control mice expressing GFP in prelimbic OTRCs were exposed to Contexts A and B during sham training and retrieval in the absence of conditioned or unconditioned stimuli, and the tissue was harvested 30 min after Context B exposure on Day 28. We observed robust activation of mTORC1 signaling in the prelimbic cortex during retrieval, as indicated by increased phosphorylation of ribosomal protein S6 (p-rpS6; Klann and Dever, 2004; Shrestha and Klann, 2022; Fig. 3*L*,*M*). Notably, p-rpS6 immunofluorescence was significantly elevated in prelimbic OTRCs relative to controls, indicating the recruitment of oxytocin-responsive cells during remote DSAA memory retrieval (Fig. 3*L*,*N*).

Collectively, these data establish that additional reinforcement does not strengthen but instead degrades safety cue discrimination at a remote time point, identifying extended training as a boundary condition that promotes maladaptive generalization of active avoidance.

### Coemergence of avoidance and freezing as complementary defensive responses during high threat

To define the dynamic range of DSAA memory, we systematically varied the intensity of the footshock US, delivering 0.1 mA (weak), 0.2 mA (medium; standard DSAA), or 0.4 mA (strong) shocks to separate cohorts of mice while holding all other parameters constant (Fig. 4*A*). This design allowed us to map the paradigm's operating range and to test whether threat magnitude governs the transition from discriminative avoidance to generalization. We used the naive DSAA protocol, in which animals infer tone-shock and tone-behavior contingencies through Bayesian updating under initial uncertainty. During training, all groups exhibited robust AR to the threat-predictive cue (CS+). However, mice in the strong US group generalized avoidance to the neutral cue (CS−; Fig. 4*B*). Avoidance latencies diverged appropriately from early to late trials across all three groups (Fig. 4*C*). The distribution of shuttling across CS+, CS−, and non-CS epochs remained stable, with a greater proportion of shuttling occurring during CS+ presentations (Fig. 4*D*), and total shuttling did not differ between groups (Fig. 4*E*). In contrast, freezing responses scaled with US intensity (Fig. 4*F*). The weak US group displayed minimal freezing to either cue. The medium US group showed a selective increase in freezing to the CS− during training, paralleling changes in avoidance latency. Strikingly, mice exposed to the strong US exhibited a pronounced escalation of freezing to both CS+ and CS−. Importantly, this loss of freezing discrimination occurred despite preserved discrimination in avoidance latency during training in the high-threat group.

**Figure 4.**
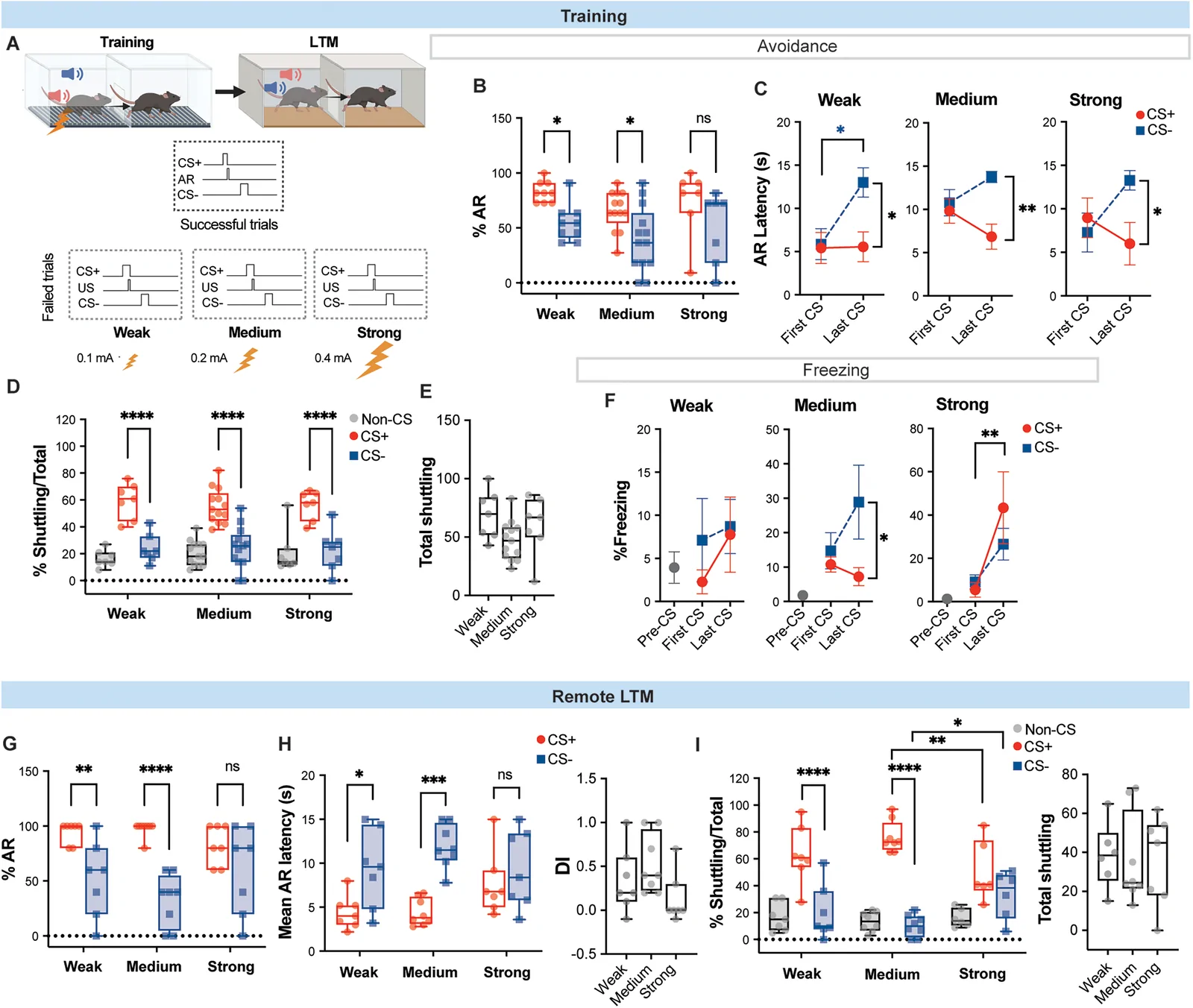
***A***, Schematic illustrating modulation of US intensity to define the dynamic range of DSAA learning and memory. Successful trials were defined identically across groups: shuttling during the CS+ prevented delivery of the footshock US. Failed trials (no shuttling during CS+) resulted in delivery of a weak (0.1 mA), medium (0.2 mA), or strong (0.4 mA) US, corresponding to group assignment. ***B***, During DSAA training, differential AR were evident in the weak and medium US groups but were diminished in the strong US group. ***C***, All three groups (weak, medium, and strong US) exhibited differential avoidance latency learning from the first to the last CS presentation. ***D***, Distribution of shuttling behavior (expressed as percentage of total shuttling) across CS+, CS−, and non-CS epochs for weak, medium, and strong US groups during training. ***E***, Total shuttling during DSAA training. ***F***, Freezing responses during pre-CS, first CS, and last CS epochs. Freezing to CS− escalated in both the medium and strong US groups, while freezing to CS+ also increased in the strong US group. ***G***, During remote LTM testing, differential AR were maintained in the weak and medium US groups, whereas the strong US group exhibited memory generalization. ***H***, Avoidance memory generalization was evident in the strong US group, while robust discrimination was preserved in the weak and medium US groups. ***I***, Left, Distribution of shuttling behavior (percentage of total shuttling) across CS+, CS−, and non-CS epochs for weak, medium, and strong US groups during remote LTM. Right, Total shuttling across groups. Statistical analyses: ***B***, ***D***, ***G***, ***H***, ***I***, Two-way ANOVA with Bonferroni’s post hoc test; ***C***, ***F***, RM two-way ANOVA with Bonferroni’s post hoc test; panels ***E***, ***H*** (right), ***I*** (right), one-way ANOVA. Sample size, *n* = 6–13 mice per group. *p* < 0.05; *p* < 0.01; *p* < 0.001; *p* < 0.0001; ns, not significant. Further characterization of footshock intensity effects on cue discrimination, including generalized responding in the weak and strong US groups, is provided in Extended Data Figure 4-1.

10.1523/ENEURO.0123-26.2026.f4-1 Figure 4-1 **A)** Box-and-whisker plots depicting differential avoidance responses (AR) in weak, medium, and strong US groups during the recent long-term memory (LTM) test. The medium US group exhibited significantly higher ARs to the CS + compared to the CS−, indicating preserved discrimination. In contrast, both weak and strong US groups displayed generalized responding across cues. **4-1B)** Left: Mean AR latency revealed cue discrimination in the medium US group, whereas latency responses were generalized in the weak and strong US groups. Right: The discrimination index did not differ significantly among the three groups. **4-1C)** Left: Shuttling distribution demonstrated appropriate divergence toward the CS + relative to the CS− and non-CS epochs in the medium and strong US groups; this divergence was absent in the weak US group. Right: Total shuttling did not differ across groups. **4-1D)** During the recent LTM test, freezing responses were higher to the CS + than the CS−, with group-dependent differences in CS−-evoked freezing. **4-1E)** At remote LTM, the strong US group showed significantly elevated freezing to both CS + and CS− compared to the weak and medium US groups, indicating enhanced generalized defensive responding. **Statistical analyses**: 4-1A, 4-1B-left, 4-1C-left, 4-1D, 4-1E) Two-way ANOVA with Bonferroni post hoc test; 4-1B-right, 4-1C-right) One-way ANOVA. **Sample siz**e: n = 6-13 mice per group. p < 0.05, p < 0.01, p < 0.001, p < 0.0001; ns, not significant. Download Figure 4-1, TIF file.

We next evaluated recent and remote LTM at 24 h and 28 d after training, respectively. At the recent LTM time point, both the weak and strong US groups showed poor CS+/CS− discrimination, whereas the medium US group maintained robust separation between the two cues (Extended Data Fig. 4-1*A*,*B*). Critically, these two failures had opposite origins: the weak US group's convergent responding reflected weak overall learning—shuttling distributions across CS+, CS−, and non-CS epochs converged rather than diverging, indicating poor memory retention despite comparable total shuttling—whereas the strong US group's convergence reflected genuine overgeneralization, with robust avoidance now extended to the safety cue (Extended Data Fig. 4-1*C*). Cue-evoked freezing remained low in the weak and medium US groups but was elevated and nondiscriminative in the strong US group (Extended Data Fig. 4-1*D*). At the remote LTM time point, the strong US group continued to show generalized AR and latencies, whereas the weak and medium US groups responded differentially to CS+ versus CS− (Fig. 4*G*,*H*). Consistently, shuttling distributions diverged across CS+, CS−, and non-CS epochs in the weak and medium groups but converged in the strong (high-threat) group; as a proportion of total shuttling, CS+-directed shuttling was reduced, and CS−-directed shuttling increased in the strong relative to the medium US group (Fig. 4*I*). Freezing remained elevated to both CS+ and CS− in the high-threat group relative to the others at remote LTM (Extended Data Fig. 4-1*E*). Together, these results define an inverted-U relationship between threat magnitude and discrimination: insufficient threat yields weak, poorly retained memory, excessive threat drives maladaptive generalization of avoidance to the safety cue, and only intermediate threat supports stable, discriminative avoidance memory. Notably, the high-threat condition additionally elevated freezing to both cues, suggesting that these animals recruit complementary defensive responses in which cue-evoked freezing precedes instrumental shuttling. This dynamic range provides the rationale for our standard 0.2 mA intensity consistent with previous studies(Campese et al., 2016; Tsutsui-Kimura et al., 2017; Capuzzo and Floresco, 2020): the medium US was sufficiently aversive to drive robust active avoidance while preserving the selection of active (shuttling) over passive (freezing) defensive responses, whereas increasing intensity to 0.4 mA collapsed cue discrimination and recruited generalized freezing. Standard-condition intensities were therefore chosen to remain within the range that supports discriminative active avoidance rather than a generalized defensive state.

### Sex-specific generalization of cued avoidance memory in a TSC mouse model

Having established that threat-safety discrimination operates within a defined behavioral range, we next asked whether it is vulnerable to genetic perturbation of the oxytocin receptor circuitry implicated in prefrontal discrimination. We recently developed a mouse model of emotional dysregulation in tuberous sclerosis complex (TSC), a neurodevelopmental disorder associated with a broad spectrum of neuropsychiatric symptoms, including anxiety (Tabaka et al., 2025). In this model, constitutive deletion of one Tsc2 allele in oxytocin receptor-expressing (OTR^+^) cells (Fig. 5*A*) produces sex-specific vulnerability to social isolation in adulthood and disrupts innate avoidance across assays including the open field, social approach, and marble burying tasks. We reasoned that learned avoidance might be similarly affected. We used OTR.Tsc2^f/+^ mice and wild-type littermate controls, analyzing males and females separately given the distinct domains of innate avoidance deficit previously observed—males impaired toward asocial targets and females toward social targets. All animals were group-housed, allowing us to test whether learned avoidance deficits emerge even without a chronic stressor such as social isolation and whether they are more sensitive to Tsc2 haploinsufficiency than innate AR.

**Figure 5.**
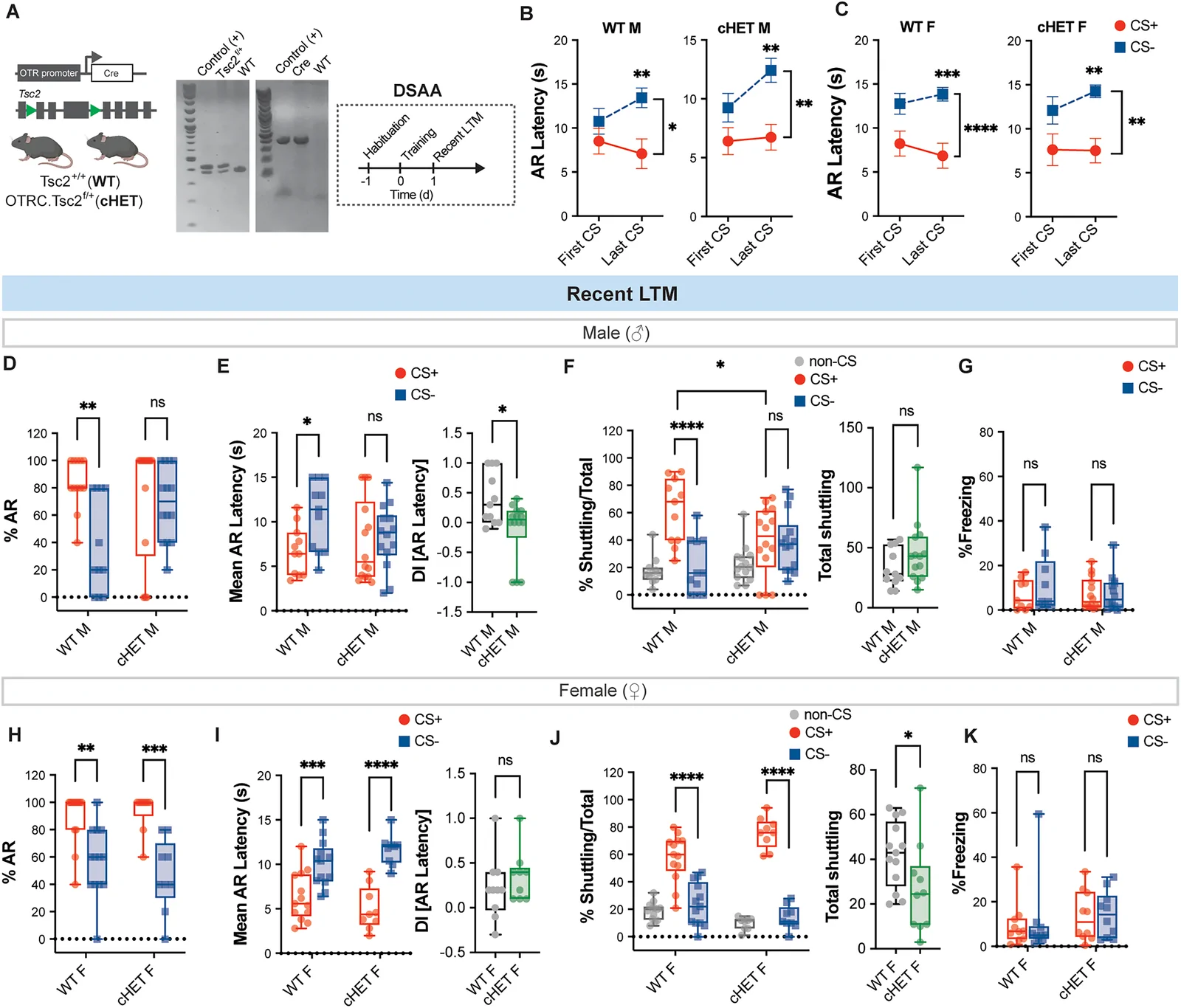
***A***, Breeding strategy used to generate OTR.Tsc2^f/+^ conditional heterozygous (cHET) mice and wild-type littermate controls. OTR-Cre transgenic mice were crossed with Tsc2^f/+^ mice. Genotyping with Cre- and Tsc2-specific primers identified offspring carrying both the Cre transgene and floxed Tsc2 allele. Both genotypes were subjected to single-session DSAA training followed by LTM testing. ***B***, Learning curves depicting changes in avoidance latency from the first to the last CS presentation for each cue (CS+ and CS−) in males of both wild-type and cHET strains. ***C***, Learning curves depicting changes in avoidance latency from the first to the last CS presentation for each cue (CS+ and CS−) in females of both wild-type and cHET strains. ***D***, During recent LTM testing, wild-type males exhibited robust differential avoidance to CS+ versus CS−, whereas cHET males failed to discriminate between cues. ***E***, Left, Mean avoidance latencies during recent LTM in wild-type and cHET males. Right, Discrimination index (DI) for avoidance latency, demonstrating reduced discrimination in cHET males relative to wild-type controls. ***F***, Left, Distribution of shuttling behavior (percentage of total shuttling) across CS+, CS−, and non-CS epochs during recent LTM in wild-type and cHET males. Right, Total shuttling during recent LTM. ***G***, Cue-evoked freezing during recent LTM in male mice, which remained minimal across groups. ***H***, Female wild-type and cHET mice both displayed robust differential AR during recent LTM testing. ***I***, Left, Mean avoidance latencies for CS+ versus CS− in wild-type and cHET females. Right, DI for avoidance latency in female mice. ***J***, Left, Distribution of shuttling behavior (percentage of total shuttling) across CS+, CS−, and non-CS epochs during recent LTM in wild-type and cHET females. Right, Total shuttling during recent LTM. ***K***, Cue-evoked freezing during recent LTM in female mice, which remained minimal across genotypes. Statistical analyses: ***B***, ***C***, RM two-way ANOVA with Bonferroni’s post hoc test; Extended Data Figure 5-1*A*, *B* (left), *D*, *E* (left), two-way ANOVA with Bonferroni’s post hoc test; Extended Data Figure 5-1, *B* (right) and *E* (right), unpaired *t* test. Sample size, *n* = 10–14 mice per group. *p < 0.05; **p < 0.01; ***p < 0.001; ****p < 0.0001; ns, not significant. Further characterization of DSAA training performance in WT and OTR.Tsc2^f/+^ (cHET) male and female mice, showing comparable avoidance, shuttling, and freezing across genotypes, is provided in Extended Data Figure 5-1.

10.1523/ENEURO.0123-26.2026.f5-1 Figure 5-1 **A)** Box-and-whisker plots showing differential avoidance responses (AR) in wildtype (WT) and OTR.Tsc2^f/+^ (cHET) male mice during DSAA training. **5-1B)** Left: Shuttling distribution revealed appropriate divergence toward the CS + relative to the CS− and non-CS epochs in both WT and cHET males. Right: Total shuttling did not differ between genotypes. **5-1C)** Percent freezing during DSAA training in WT and cHET male mice, quantified during the Pre-CS baseline, first CS, and last CS epochs. **5-1D)** Box-and-whisker plots depicting differential avoidance responses (AR) in WT and OTR.Tsc2^f/+^ (cHET) female mice during DSAA training. **5-1E)** Left: Shuttling distribution showed appropriate CS + -biased divergence relative to CS− and non-CS epochs in both WT and cHET females. Right: Total shuttling did not differ between genotypes. **5-1F)** Percent freezing during DSAA training in WT and cHET female mice, measured during the Pre-CS baseline, first CS, and last CS epochs. **Statistical analyses**: 5-1F) RM Two-way ANOVA with Bonferroni post-hoc test; 5-1A, 5-1B-left, 5-1D, 5-1E-left) Two-way ANOVA with Bonferroni post hoc test; 5-1B-right, 5-1E-right) Unpaired t-test. **Sample size:** n = 10-14 mice per group. *p < 0.05, **p < 0.01, ***p < 0.001, ****p < 0.0001; ns, not significant. Download Figure 5-1, TIF file.

We subjected adult male and female mice to the naive DSAA protocol using a medium-intensity US (0.2 mA), consisting of a single training session followed by recent and remote LTM testing at 24 h and 28 d post-training, respectively. During training, all groups exhibited comparable performance, characterized by robust acquisition of differential avoidance latencies, high levels of avoidance responding, and appropriate divergence in shuttling behavior during CS+ relative to CS− and non-CS epochs (Fig. 5*B*,*C*; Extended Data Fig. 5-1*A*–*D*). Notably, cue-evoked freezing strategies diverged by sex. Males progressively increased freezing to the neutral cue (CS−) across training, whereas females showed a reduction in freezing to the threat-predictive cue (CS+) over time (Extended Data Fig. 5-1*E*,*F*). These findings point to a sex-specific allocation of defensive strategies during reinforced learning: in males, the CS+ predominantly engages instrumental avoidance, whereas the neutral CS−, despite lacking behavioral contingency, appears to become incorporated into the threat context and elicits passive freezing, potentially through coallocation within a shared temporal encoding window. Of note, we did not observe any genotype effects during training. Because acquisition of the cued AR requires perception and discrimination of the auditory cues, a hearing deficit would be expected to impair acquisition—which it did not; thus, behavioral data provide strong evidence against an auditory confound in cHET mice.

We next assessed recent LTM 24 h after training. Compared with wild-type males, OTR.Tsc2^f/+^ cHET males exhibited generalized avoidance memory, reflected in both percent avoidance and avoidance latency (Fig. 5*D*,*E*). This generalization led to a significant reduction in the discrimination index in cHET males. The distribution of shuttling responses across CS+, CS−, and non-CS epochs also converged in cHET males, indicating impaired retention of cue-specific memory in the novel context, despite normal levels of total shuttling (Fig. 5*F*). Cue-evoked freezing remained minimal in both wild-type and cHET males (Fig. 5*G*). In contrast, female cHETs displayed robust differential avoidance memory, as evidenced by appropriate discrimination in both avoidance percentage and latency (Fig. 5*H*,*I*). The distribution of shuttling responses across CS+, CS−, and non-CS epochs diverged appropriately in both wild-type and cHET females; however, total shuttling was significantly reduced in cHET females (Fig. 5*J*). Freezing responses were minimal in both groups (Fig. 5*K*). Thus, the female cHET deficit is dissociable in kind from the male phenotype, affecting overall response vigor rather than cue-specific discrimination—underscoring that the impairment in threat—safety discrimination is male-specific.

To determine whether the generalized avoidance observed in cHET males reflected inadequate discrimination learning after a single training session, we extended training by two additional days and assessed remote LTM 28 d later. Additional training enhanced overall avoidance performance and reduced latencies across genotypes (Fig. 6*A*,*B*). Wild-type males retained robust discrimination between CS+ and CS−, as evidenced by differential AR and latencies. In contrast, cHET males continued to generalize between the cues (Fig. 6*C*,*D*). Consistent with this, the distribution of shuttling responses across CS+, CS−, and non-CS epochs remained convergent in cHET males but diverged appropriately in wild-type controls, despite comparable total shuttling between groups (Fig. 6*E*). Freezing responses remained minimal in both groups (Fig. 6*F*).

**Figure 6.**
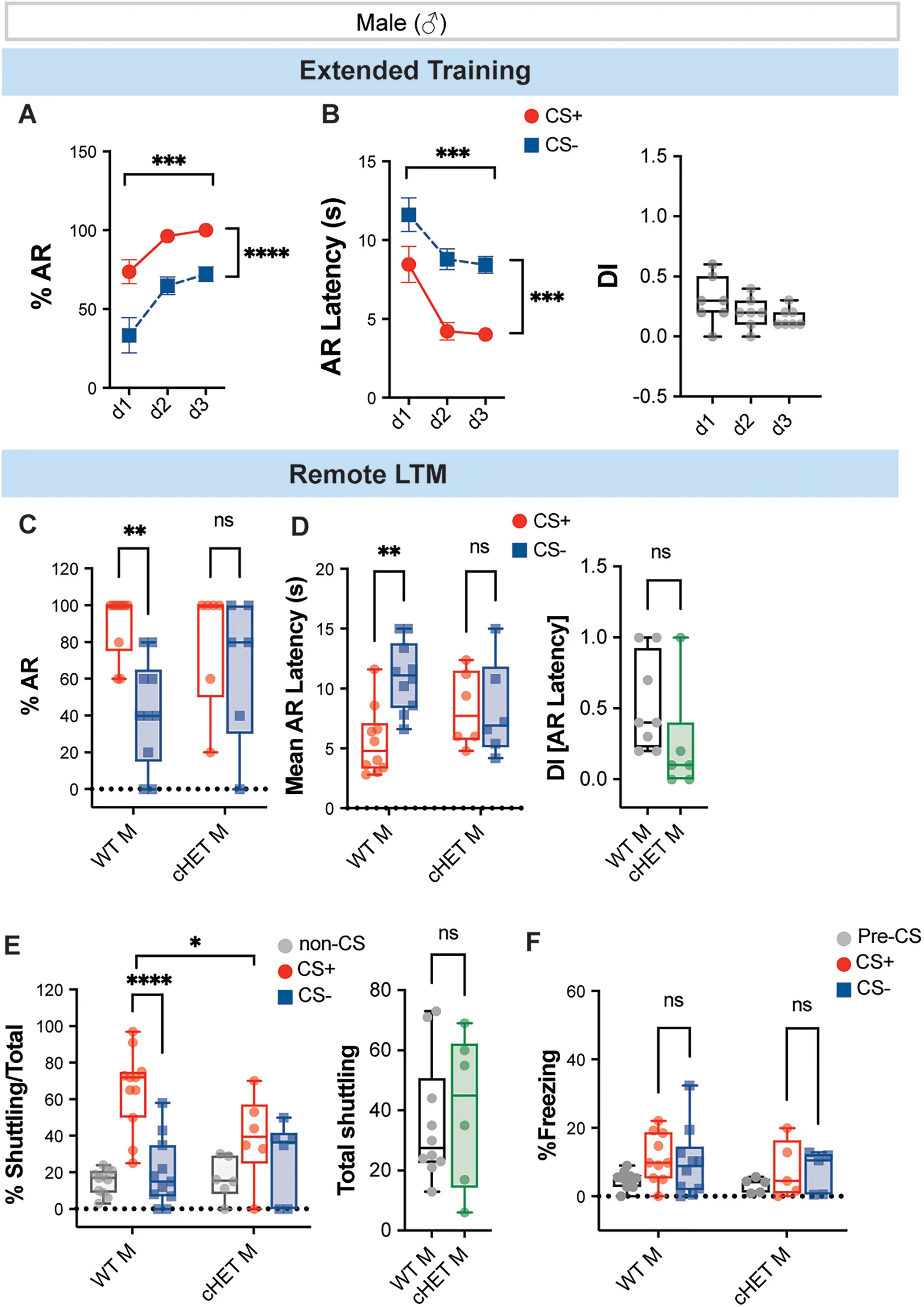
***A***, *X*–*Y* plots illustrating the learning curve for AR to CS+ and CS− across successive days of training (overtraining) in OTR.Tsc2^f/+^ (cHET) male mice. ***B***, Left, Avoidance latency progressively decreased over additional training days in cHET males, indicating improved task performance. Right, The discrimination index remained stable across all three training days. ***C***, During remote LTM testing, overtrained wild-type (WT) males exhibited clear differential avoidance between CS+ and CS−, whereas overtrained cHET males displayed generalized responding across cues. ***D***, Mean avoidance latency at remote LTM reflected preserved cue discrimination in WT males but generalized latency responses in cHET males. ***E***, Shuttling distribution demonstrated appropriate CS+-biased divergence relative to CS− and non-CS epochs in overtrained WT males; in contrast, distributions converged in cHET males despite comparable total shuttling between genotypes. ***F***, Cue-evoked freezing remained minimal for both CS+ and CS− during remote LTM in overtrained WT and cHET males. Statistical tests: ***A***, ***B***, left, RM two-way ANOVA with Bonferroni’s post hoc test; ***B***, right, one-way ANOVA; ***C***, ***D*** (left), ***E*** (left), ***F***, Two-way ANOVA with Bonferroni’s post hoc test; ***D*** (right), ***E*** (right), unpaired *t* test. Sample size, *n* = 5–10 mice per group. *p < 0.05; **p < 0.01; ***p < 0.001; ****p < 0.0001; ns, not significant.

Together, these findings indicate that male OTR.Tsc2^f/+^ cHET mice exhibit a specific impairment in the consolidation and/or retrieval of associative cue-evoked avoidance memory rather than a deficit in task acquisition or locomotor output. This behavioral phenotype is consistent with the reduction in nascent protein synthesis observed in medial prefrontal OTRCs, a molecular process critical for LTM consolidation (Tabaka et al., 2025). Moreover, the engagement of the mTORC1 pathway in prefrontal OTRCs during memory retrieval suggests that sustained translational control within these cells supports persistent cellular plasticity required for accurate discrimination between threat-predictive and neutral cues.

## Discussion

Although conventional SAA and DSAA produced comparable acquisition during training, only DSAA strengthened recent retrieval, yielding higher avoidance rates, shorter response latencies, and reduced freezing. Discrimination during training strongly predicted discrimination at retrieval, and avoidance probability closely tracked latency reductions, indicating consolidation of a temporally precise cue-action association. Notably, this enhancement did not depend on prior pavlovian conditioning. Naive animals inferred contingencies within a single session, suggesting that differential cue structure itself facilitates Bayesian updating under uncertainty. The neutral cue therefore appears to reduce ambiguity during encoding, sharpening consolidation of CS+-specific instrumental memory.

More broadly, incorporation of a neutral cue into the avoidance paradigm enhances consolidation and long-term expression of cue-specific memory without altering acquisition dynamics. Why the neutral safety cue facilitates consolidation remains an open question. One possibility is that it engages flexible internal models formed in the mPFC, which link environmental stimuli to aversive outcomes—even when associations are indirect (Barron et al., 2020; Gu and Johansen, 2025). The inclusion of a neutral cue may enrich the representational structure of the learning environment, promoting more robust inferential encoding and stabilization of memory traces. Inferential memory representations are thought to emerge through multistep encoding mechanisms involving recruitment and stabilization of mPFC neurons, particularly those projecting to the amygdala. This framework may explain why the CS− transiently evokes defensive freezing during training, which diminishes across sessions yet persists at remote time points in high-threat conditions. At the same time, differential avoidance memory remains highly sensitive to boundary conditions, including extended training, heightened threat intensity, and genetic disruption of translational plasticity.

The single-session nature of DSAA may also depend on species-specific defensive biases. Defensive strategy selection is influenced more strongly by body size than by sex or age: smaller animals preferentially adopt proactive avoidance, whereas larger animals tend toward reactive freezing (Laughlin et al., 2020). Thus, the efficiency of single-session DSAA may be particularly suited to mice, which are substantially smaller than Long–Evans rats (Laughlin et al., 2020). In contrast, previous rat studies required prolonged cue durations (up to 240 s), extensive reinforcement (30 trials per session), and multiday training to enhance avoidance. In our protocol, a modest 15 s CS+ presented only 11 times, interleaved with a temporally distinct CS−, was sufficient to generate robust LTM. Importantly, DSAA does not include an explicit feedback signal following the AR. Animals infer action value solely through omission of the footshock. Because the CS− was never reinforced in any animal, regardless of CS+ performance, its objective contingency was identical across subjects, and its 20–90 s separation from the AR precludes a feedback role. Across individuals, however, CS+ and CS− avoidance were positively correlated, with the strongest CS+ avoiders also responding most to the CS−, while cue discrimination declined at the trend level as CS+ avoidance rose. We interpret this covariation as a shared, graded avoidance propensity across the cohort rather than a failure to acquire the CS+ contingency, which would instead predict elevated or erratic CS− responding among the poorest CS+ performers. Under standard and extended-training conditions, neither CS+ nor CS− evokes substantial freezing during retrieval, indicating suppression of central amygdala-periaqueductal gray circuits in favor of proactive avoidance. Only high-threat conditions produce coexpression of freezing and avoidance, accompanied by loss of cue discrimination. Manipulating shock intensity further revealed that discrimination operates within an optimal threat range. Standard (0.2 mA) footshock produced the sharpest divergence in AR, whereas both weaker and stronger shocks reduced response precision. These findings are consistent with Mowrer's two-factor theory (Mowrer and Lamoreaux, 1946; Fernández-Teruel and Tobeña, 2020): avoidance and freezing are goal-directed behaviors serving to reduce fear, but their balance depends on the arousal level.

At the circuit level, remote DSAA retrieval robustly engaged prefrontal OTRCs and activated mTORC1-dependent translational signaling, as indicated by increased phosphorylation of ribosomal protein S6 (von Manteuffel et al., 1997). These findings suggest that retrieval of discriminative avoidance memory recruits protein synthesis-dependent plasticity within oxytocin-sensitive prefrontal ensembles. Retrieval is therefore not a passive readout but requires sustained translational control to stabilize or reactivate cue-specific neural representations. This aligns with models in which oxytocin signaling constrains anxiety-related responding and promotes discrimination between threat and safety within limbic–prefrontal circuits (Sabihi et al., 2014; Triana-Del Rio et al., 2022). However, the current design cannot fully dissociate retrieval-associated translational signaling from tone-evoked or motor-associated activation and identifying phase-resolved OTRC profiling as an important direction for future work. Tsc2 haploinsufficiency in OTR-expressing cells produced a male-specific impairment in associative avoidance memory. Male OTR.Tsc2^f/+^ cHET mice displayed intact acquisition but persistent generalization at both recent and remote time points, a deficit not rescued by additional training and not attributable to altered locomotion or freezing. Given that TSC2 regulates both mTORC1- and PERK-eIF2α–dependent translational control (Ehninger et al., 2008; Tyagi et al., 2015; Saré et al., 2018; Tabaka et al., 2025), these findings implicate disrupted protein synthesis in the failure to consolidate or retrieve cue-specific memory. Together, the behavioral and molecular data support a model in which oxytocin-modulated translational control in the emotional memory network gates the balance between discrimination and generalization. When this regulatory axis is compromised—by excessive threat or impaired TSC signaling—precise avoidance encoding collapses into generalized defensive responding, providing a mechanistic framework for safety-learning deficits in neurodevelopmental and stress-related disorders. Considering the well-established role of PVN oxytocin neurons in stress buffering, a generalization phenotype could in principle arise from impaired buffering—an exaggerated stress state biasing behavior toward overgeneralized responding—rather than from altered avoidance encoding per se. We note the distinction that our genetic manipulation targets Tsc2 in OTR-expressing (receptor) cells, not OT-producing PVN neurons; nonetheless, we concede that downstream or network-level effects on stress-regulatory circuits cannot be excluded on the basis of the present data, and we identify dissection of PVN involvement as a valuable direction for future work.

## Data Availability

All source data supporting the findings are available upon request. No custom scripts were generated for data analysis. Custom pipelines for data analysis are available from the corresponding author upon reasonable request.

## Synthesis

Reviewing Editor: Katrina Choe, McMaster University Decisions are customarily a result of the Reviewing Editor and the peer reviewers coming together and discussing their recommendations until a consensus is reached. When revisions are invited, a fact-based synthesis statement explaining their decision and outlining what is needed to prepare a revision will be listed below. The following reviewer(s) agreed to reveal their identity: Jacco Renstroem. Note: If this manuscript was transferred from JNeurosci and a decision was made to accept the manuscript without peer review, a synthesis may not be available.

The manuscript provides interesting insights into the mechanistic processes underlying flexible threat discrimination and its long-term retrieval using a modified signal active avoidance paradigm. Identification of the role of prefrontal oxytocinergic activity in long-term fear discrimination could be a valuable contribution to the field, lending itself well to future work investigating pathological models of fear overgeneralization. Reviewers commented that the manuscript is generally well written, figures are clear and well designed, and overall, the report provides interesting insights into the active avoidance and fear generalization under various contexts. However, the reviewers shared several key concerns with the current report, detailed below.

1) The manuscript should clarify how safety signals are experienced across animals with differing task performance. If safety signals are presented even when mice fail to avoid shock, animals may form fundamentally different associations with the cue depending on their success rate. Mice that reliably avoid may interpret the cue as a true safety signal, whereas poor performers may associate it differently or inconsistently. This raises concerns regarding interpretation of learning mechanisms and comparability across subjects.

2)The rationale for performing the Pavlovian conditioning and the "overtraining" conditions in conjunction with the DSAA can be better explained. It is currently challenging to understand what motivated the inclusion of these specific conditions from these statements: "to test whether priors influence.. (line 107)", "whether additional training modifies.. (line 141)", and "to define the dynamic range .. (line 181).

3) It is unclear why data presented in the supplemental material was not incorporated more prominently into the main figures, despite its apparent relevance to the primary findings, especially data pertaining to remote LTM. Consider moving key supplemental results into the main figures to improve clarity and accessibility.

4) Fig 3, the "medium" shock condition dataset appears to be identical to the data presented in Fig 1 ("naïve") and Fig 2 ("single episode"). The authors should either avoid using the same datasets or clearly state in the manuscript if the same data are represented across multiple figures.

5)Increased phosphorylation of rpS6 is observed in oxytocin receptor expressing cells in the medial prefrontal cortex, and is attributed to memory retrieval; however, it is still possible that the increased expression is associated with tone presentations or the running part of the escape response, not purely due to the retrieval. The experiment should be better controlled than "box only", to make this statement, or the limitations should be stated in the discussion. Also, it is uncertain whether these neurons are recruited during the training and recent LTM phases. Again it would be useful to further discuss these highly relevant points.

6) The use of the OT-specific Tsc2 knockout model is interesting and potentially important, but the introduction would benefit from additional background explaining the rationale for selecting this model apart from availability. In particular, the relationship between OT-specific Tsc2 deletion and anxiety-related phenotypes should be developed more clearly to support the broader conceptual framework of the study.

7) Statistical reporting throughout the manuscript is insufficient. At minimum, complete ANOVA tables (including degrees of freedom, F-values, p-values, and effect sizes) should be provided in supplemental material. This is especially important given the relatively low sample sizes and the appearance that several effects may be strongly influenced by potential outliers.

8) The use of the term "overtraining" is not fully justified. Typically, overtraining implies performance has reached a stable ceiling or asymptotic level. However, the behavioral data shown thus far appear highly variable and do not clearly demonstrate ceiling performance. A more conservative term such as "extended" or "repeated" DSAA training may be more appropriate unless additional evidence is provided.

9) The term "episode" could be interpreted as referring to an individual trial. Using "single-day" or similar terminology may improve clarity and reduce ambiguity. Moreover, the rationale for selecting 11 presentations per session should be explained. As currently written, the choice appears arbitrary.

10) One could argue that reported neural activation patterns shown in Figure 2 may simply reflect exposure to electric shock versus no stimulus rather than processes specific to avoidance learning or safety signaling. Additional colocalization analyses (e.g., with OT neurons) would strengthen interpretation of the observed activity patterns.

11) A major oxytocin-related region not discussed is the paraventricular nucleus (PVN), whose OT neurons are strongly activated by stress and are implicated in stress buffering. The observed phenotype could potentially reflect impaired stress buffering within PVN circuits, resulting in exaggerated and thus overgeneralized stress responses rather than altered avoidance learning per se. This possibility should be discussed.

12) The footshock intensities used appear lower than those commonly employed in the literature; further rationale for selecting these parameters should be provided.

13) Why was remote LTM not assessed after a single trial in cHET mice?

14) Auditory threshold was assessed in WT mice, but not in cHET mice later. It would be prudent to evaluate or discuss if cHET mice have any auditory abnormalities compared to WT that may alter the experience of auditory stimuli.

Minor

• Results: line 107, unsure exactly when the Pavlovian conditioning was performed. Is it the same day as the DSAA? How long before the training?

• Methods: line 491, the sound intensity level of the white noise machine should be stated.

• Figure 1C) labelled both CS as CS+. Also, it is unclear why some bars CS in C are smaller than others. I would suggest adding dashed vertical lines to indicate separation of epochs.

• Figure 1 legend does not match the graphs shown. Correlation graph is 1L whereas freezing graph is 1M (not mentioned here.)

• Figure 4K legend is incorrect, 4K is missing and J does not match the graph. Furthermore, 4G and K should have equal number of y-axis ticks, and consider standardizing Y axis to make comparison easier.

• Several grammatical inconsistencies throughout. Please check fonts and sizes for + and -; differentiation between hyphens, em- and en-dashes throughout; use of oC instead of {degree sign}C; spaces between commas.

• Figure 4F coronal schematic is currently blank. Should this include an injection size? Otherwise, the inclusion is obsolete.

## Author Response

July 22, 2026 We thank the editor and reviewers for their constructive evaluation of our manuscript, and for their positive assessment of the writing, figures, and conceptual contribution. The comments have substantially improved the clarity and rigor of the work. Below we respond to each point individually; reviewer comments are shown in italics and our responses in plain text. The updated sections in the manuscript are highlighted in yellow.

Major Comments 1) The manuscript should clarify how safety signals are experienced across animals with differing task performance. If safety signals are presented even when mice fail to avoid shock, animals may form fundamentally different associations with the cue depending on their success rate. Mice that reliably avoid may interpret the cue as a true safety signal, whereas poor performers may associate it differently or inconsistently. This raises concerns regarding interpretation of learning mechanisms and comparability across subjects.

We thank the reviewer for this important point, which prompted an analysis that we believe both clarifies the paradigm and directly tests the proposed mechanism. First, a structural feature of the task constrains the concern: the CS- is never reinforced for any animal, regardless of CS+ performance, so its objective contingency is identical across all subjects. It is additionally dissociated in time from the avoidance response by 20-90 s and therefore does not act as a feedback signal. We have moved this clarification forward into the Results and Methods [Line 348, Line 222].

Second, and more directly, we tested the specific hypothesis the reviewer raises. If reliable avoiders interpreted the CS- as a true safety signal while poor performers formed a different association, one would predict CS- responding to be suppressed in high CS+ performers - i.e., a negative relationship between CS+ and CS- avoidance. We instead observe a significant positive correlation between % avoidance to CS+ and % avoidance to CS- (new Supplementary Fig. 1B; r2 = 0.501, p = 0.0068**). Animals with the highest CS+ avoidance also showed the highest CS- responding. This is inconsistent with a mechanism in which CS- responding is driven by CS+ failure or by inconsistent association in poor performers; rather, residual CS- responding co-varies with a shared, graded avoidance propensity across the cohort. Consistent with this interpretation, the discrimination index showed a trend-level decline as CS+ avoidance increased (new Supplementary Fig. 1C; r² = 0.249, p = 0.083).

We therefore interpret individual variation in CS- responding not as a categorical difference in how successful versus unsuccessful animals construe the safety cue, but as a continuous generalization gradient scaling with overall avoidance drive. This individual-level relationship parallels, and extends, the central thesis of the manuscript: discrimination operates within a defined dynamic range and degrades as defensive drive rises - whether that drive is elevated experimentally (high-threat and extended-training conditions) or reflects baseline individual differences. We have added these analyses as a new supplementary figure, and we have revised the Results (Line 348) and Discussion (Line 583) to state explicitly that discrimination is not uniform across individuals and to frame this graded, drive-linked generalization as consistent with the proposed boundary conditions on cue-specific avoidance memory.

2) The rationale for performing the Pavlovian conditioning and the "overtraining" conditions in conjunction with the DSAA can be better explained. It is currently challenging to understand what motivated the inclusion of these specific conditions from these statements: "to test whether priors influence.. (line 107)", "whether additional training modifies.. (line 367)", and "to define the dynamic range .. (line 181).

We agree that the motivation for each experimental arm should be stated explicitly, and we have added a framing sentence to the opening of each corresponding Results section: • Priors / Pavlovian trial: added to test whether single-session consolidation requires explicit tone-shock instruction or instead emerges from contingency inference under initial uncertainty. The finding that naïve animals matched the priors group is, we believe, one of the more notable results, and this framing makes its significance clear. This has been added in Results section (Line 367) • Extended training group (renamed per Comment 8): added to determine whether additional reinforcement strengthens cue specificity or, conversely, degrades it - a boundary condition with direct relevance to compulsive/rigid avoidance (Line 404). • US-intensity group: added to map the dynamic range of the paradigm and test whether threat magnitude governs the transition from discriminative avoidance to generalization (Line 451).

3) It is unclear why data presented in the supplemental material was not incorporated more prominently into the main figures, despite its apparent relevance to the primary findings, especially data pertaining to remote LTM. Consider moving key supplemental results into the main figures to improve clarity and accessibility.

We appreciate this suggestion and have reorganized the figures accordingly. Specifically: • The SAA-vs-DSAA recent-LTM divergence (formerly Supplementary Fig. 1F-I), which establishes the foundational rationale for the safety-cue manipulation, has been promoted to main Figure 1. • The extended-training remote-LTM cHET data (formerly Supplementary Fig. 6), which supports the central claim that the male-specific deficit is not rescued by additional training, has been promoted to main Figure 6.

We have retained in the supplement the control analyses and secondary time points that support but do not carry the primary narrative, to keep the main figures legible. We hope this reorganization improves accessibility while preserving figure clarity.

4) Fig 3, the "medium" shock condition dataset appears to be identical to the data presented in Fig 1 ("naïve") and Fig 2 ("single episode"). The authors should either avoid using the same datasets or clearly state in the manuscript if the same data are represented across multiple figures.

The reviewer is correct. The naïve DSAA group (Fig. 1 -> now Fig. 2) and the single-session group (Fig. 2 -> now Fig. 3), and the medium-US group (Fig. 3 -> now Fig. 4) represent a single shared reference cohort, as these conditions are experimentally identical (naïve protocol, 0.2 mA US, single training session) and serve as the common comparator against which the priors, extended-training, and US-intensity manipulations are evaluated. This is a deliberate and, we believe, appropriate use of a reference group; our error was in not stating it. We have now added an explicit statement in the Methods that this cohort is shared across Figures 2-4 [Line 251].

5) Increased phosphorylation of rpS6 is observed in oxytocin receptor expressing cells in the medial prefrontal cortex, and is attributed to memory retrieval; however, it is still possible that the increased expression is associated with tone presentations or the running part of the escape response, not purely due to the retrieval. The experiment should be better controlled than "box only", to make this statement, or the limitations should be stated in the discussion. Also, it is uncertain whether these neurons are recruited during the training and recent LTM phases. Again it would be useful to further discuss these highly relevant points.

We thank the reviewer for this rigorous point. We agree that the box-only control accounts for context exposure, handling, and time-of-euthanasia, but does not by itself isolate retrieval-associated activity from tone-evoked or motor-associated activity.

While a dedicated tone/locomotion control was beyond the scope of the present study, we note that the box-only comparison is not shock-versus-nothing: box-only animals were exposed to both Context A and Context B and to the full handling and locomotor demands of the sessions, differing from DSAA animals principally in the presence of the learned cue-outcome contingency. We have nonetheless added a limitations paragraph to the Discussion (Line 605) explicitly acknowledging that the current design cannot fully dissociate retrieval-associated translational signaling from tone-evoked or motor-associated activation and identifying phase-resolved OTRC profiling as an important direction for future work.

6) The use of the OT-specific Tsc2 knockout model is interesting and potentially important, but the introduction would benefit from additional background explaining the rationale for selecting this model apart from availability. In particular, the relationship between OT-specific Tsc2 deletion and anxiety-related phenotypes should be developed more clearly to support the broader conceptual framework of the study.

We have expanded the Introduction to build an explicit, hypothesis-driven rationale for the model (Line 86). Drawing on our prior characterization of this mouse strain (Tabaka et al., 2025) and the surrounding literature, we now lay out the following chain: prefrontal OTR-expressing cells disinhibit layer II/III pyramidal neurons and thereby constrain anxiety- and stress-related responses; oxytocinergic modulation of limbic-prefrontal circuits promotes discrimination between threat- and safety-predictive stimuli; and Tsc2 haploinsufficiency in these cells produces sex-specific anxiety phenotypes and disrupted innate avoidance. This motivates the specific prediction that degraded OTR-cell function should impair learned threat-safety discrimination and bias behavior toward generalization - the phenotype we test. The model is thus selected as a targeted test of this circuit hypothesis rather than for availability.

7) Statistical reporting throughout the manuscript is insufficient. At minimum, complete ANOVA tables (including degrees of freedom, F-values, p-values, and effect sizes) should be provided in supplemental material. This is especially important given the relatively low sample sizes and the appearance that several effects may be strongly influenced by potential outliers.

We have added Extended Data Table 1, a comprehensive statistics table reporting, for every comparison in the manuscript: the test used, degrees of freedom, the test statistic (F or t), the exact p-value, and sample size each keyed to its figure panel.

8) The use of the term "overtraining" is not fully justified. Typically, overtraining implies performance has reached a stable ceiling or asymptotic level. However, the behavioral data shown thus far appear highly variable and do not clearly demonstrate ceiling performance. A more conservative term such as "extended" or "repeated" DSAA training may be more appropriate unless additional evidence is provided.

We agree. Our data do not demonstrate asymptotic/ceiling performance, and the term "overtraining" implies a plateau we did not establish. We have replaced "overtraining"/"overtrained" with "Extended-Training" throughout the manuscript, figures, and legends.

9) The term "episode" could be interpreted as referring to an individual trial. Using "single-day" or similar terminology may improve clarity and reduce ambiguity. Moreover, the rationale for selecting 11 presentations per session should be explained. As currently written, the choice appears arbitrary.

To remove ambiguity, we have defined "single-episode" explicitly at first use as referring to a single session (not a single trial), and we have ensured the usage is consistent throughout.

Regarding the number of CS+ presentations, we have added a Supplementary Figure 1A and justification to the Results section (Line 339).

The number of CS+ presentations was chosen based on our previously validated threat-discrimination protocol (Shrestha et al, 2020), in which three presentations each of the CS+ and CS- within a single session were sufficient to establish a discriminative reactive freezing memory. Under DSAA, however, mice failed to discriminate between the two stimuli through the first five CS+/CS- presentations, despite the tone-shock contingency for the CS+. Discrimination emerged only after more than ten presentations: by the eleventh presentation, avoidance latencies for the two cue types differed significantly (Supplementary Fig. 1A). Because the total session duration remained approximately 27 min - still within the bounds of single-session DSAA - we adopted the 11-CS protocol for all subsequent studies. This regimen was sufficient to drive robust consolidation while remaining below the extended-training range that we subsequently found to degrade cue specificity.

10) One could argue that reported neural activation patterns shown in Figure 2 may simply reflect exposure to electric shock versus no stimulus rather than processes specific to avoidance learning or safety signaling. Additional colocalization analyses (e.g., with OT neurons) would strengthen interpretation of the observed activity patterns.

We thank the reviewer and would clarify two points. First, our p-rpS6 quantification is already performed within the receptor-defined OTR population: p-rpS6 immunofluorescence was measured specifically within GFP-labeled OTR-expressing cells (Fig. 3N), which constitutes colocalization with the receptor-defined ensemble of interest. We have revised the text and legend to make this explicit. We note a possible source of confusion between OTR-expressing (receptor) cells - the population we target and quantify - and OT-producing (peptidergic PVN) neurons; we now distinguish these clearly (see also Comment 11).

Second, regarding the shock-versus-no-stimulus concern, this overlaps with Comment 5, and we address them together: the box-only control experienced both contexts and the full session structure, so the relevant comparison is trained-retrieval versus context-exposure rather than shock versus nothing.

11) A major oxytocin-related region not discussed is the paraventricular nucleus (PVN), whose OT neurons are strongly activated by stress and are implicated in stress buffering. The observed phenotype could potentially reflect impaired stress buffering within PVN circuits, resulting in exaggerated and thus overgeneralized stress responses rather than altered avoidance learning per se. This possibility should be discussed.

We appreciate this thoughtful alternative interpretation and have added a paragraph to the Discussion addressing it (Line 618). We acknowledge the well-established role of PVN oxytocin neurons in stress buffering, and that a generalization phenotype could in principle arise from impaired buffering - an exaggerated stress state biasing behavior toward overgeneralized responding - rather than from altered avoidance encoding per se. We note the distinction that our genetic manipulation targets Tsc2 in OTR-expressing (receptor) cells, not OT-producing PVN neurons; nonetheless, we concede that downstream or network-level effects on stress-regulatory circuits cannot be excluded on the basis of the present data, and we identify dissection of PVN involvement as a valuable direction for future work.

12) The footshock intensities used appear lower than those commonly employed in the literature; further rationale for selecting these parameters should be provided.

We have added an explicit rationale in Results section (Line 484). Our intensity selection is data-supported as well as consistent with previous studies: 0.2 mA is sufficiently aversive to drive robust active avoidance while preserving the selection of active (shuttling) over passive (freezing) defensive responses. Our own dynamic-range experiment (Fig. 4) demonstrates that increasing intensity to 0.4 mA collapses cue discrimination and recruits generalized freezing. The intensities used were therefore chosen specifically to remain within the range that supports discriminative active avoidance rather than a generalized defensive state.

13) Why was remote LTM not assessed after a single trial in cHET(female) mice? We have clarified the design logic in the text. Single-session training already produced generalized avoidance memory in cHET males at the recent LTM timepoint (Fig. 5D-F). The extended-training arm was therefore designed to test the specific and clinically salient question of whether additional training could rescue this deficit at a remote timepoint - and it did not (Fig. 6). The omission of a single-session remote-LTM cHET females thus reflects a deliberate emphasis on the rescue question rather than an oversight.

14) Auditory threshold was assessed in WT mice, but not in cHET mice later. It would be prudent to evaluate or discuss if cHET mice have any auditory abnormalities compared to WT that may alter the experience of auditory stimuli.

We agree this should be addressed and would note that the behavioral data provide strong evidence against an auditory confound: cHET mice showed intact acquisition, with equivalent CS+ avoidance rates and avoidance latencies during training (Fig. 5B-C; Suppl. Fig. 5). Because acquisition of the cued avoidance response requires perception and discrimination of the auditory cues, a hearing deficit would be expected to impair acquisition - which it did not. We have added this justification to the text (Line 517).

Minor Comments 1) Results: line 107, unsure exactly when the Pavlovian conditioning was performed. Is it the same day as the DSAA? How long before the training? o Pavlovian timing (Results, line 364): We have specified when the Pavlovian trial occurred relative to DSAA training (1 hour prior).

2) Methods: line 491, the sound intensity level of the white noise machine should be stated. o White noise level (Methods, line 169): The measured sound level of the Yogasleep Dohm (58 dB at a distance of 6 ft from the machine) has been added.

3) Figure 1C) labelled both CS as CS+ . Also, it is unclear why some bars CS in C are smaller than others. I would suggest adding dashed vertical lines to indicate separation of epochs o Figure 1C: Corrected the mislabeled cue (both were labeled CS+ ; one is now CS-). We have added dashed vertical lines to demarcate each of the eleven sets of CS+ and CS-.

4) Figure 1 (now Figure 2) legend does not match the graphs shown. Correlation graph is 1L whereas freezing graph is 1M (not mentioned here.) o Figure 2 legend (L/M): Corrected the legend so that the correlation panel is 2L and the freezing panel is 2M; the freezing panel is now described in the legend.

5) Figure 4K (now Figure 5) legend is incorrect, 4K is missing and J does not match the graph. Furthermore, 4G and K should have equal number of y-axis ticks, and consider standardizing Y axis to make comparison easier. o Figure 4 legend (J/K): Corrected the panel labeling. We have standardized the number of y-axis ticks between 4G and 4K and set a common y-axis across comparable freezing panels to facilitate comparison.

6) Several grammatical inconsistencies throughout. Please check fonts and sizes for +and -; differentiation between hyphens, em- and en-dashes throughout; use of oC instead of {degree sign}C; spaces between commas. o Typography and grammar: We have performed a thorough pass correcting +/- font consistency, hyphen versus en-/em-dash usage, "oC" → {degree sign}C, comma spacing, and other grammatical inconsistencies.

7) Figure 4F coronal schematic is currently blank. Should this include an injection size? Otherwise, the inclusion is obsolete. o Figure 4F coronal schematic: We have removed the panel.

We thank the reviewers again for their thorough and constructive comments, which have meaningfully strengthened the manuscript. We hope the revised version is now suitable for publication and look forward to your response.
